# Macrophage innate immune responses delineate between defective translocon assemblies produced by *Yersinia pseudotuberculosis* YopD mutants

**DOI:** 10.1080/21505594.2023.2249790

**Published:** 2023-08-24

**Authors:** Salah I. Farag, Monika K. Francis, Jyoti M. Gurung, Sun Nyunt Wai, Hans Stenlund, Matthew S. Francis, Aftab Nadeem

**Affiliations:** a Department of Molecular Biology, Umeå University, Umeå, Sweden; b Umeå Centre for Microbial Research, Umeå University, Umeå, Sweden; c The Laboratory for Molecular Infection Medicine Sweden, Umeå University, Umeå, Sweden; d Department of Plant Physiology, Umeå Plant Science Centre (UPSC), Umeå University, Umeå, Sweden; e Swedish Metabolomics Centre (SMC), Umeå, Sweden

**Keywords:** Cytokine profiling, inflammasome, programmed cell death, anti-phagocytosis, translocon complexes, bacteria-eukaryotic cell contact

## Abstract

Translocon pores formed in the eukaryotic cell membrane by a type III secretion system facilitate the translocation of immune-modulatory effector proteins into the host cell interior. The YopB and YopD proteins produced and secreted by pathogenic *Yersinia* spp. harboring a virulence plasmid-encoded type III secretion system perform this pore-forming translocator function. We had previously characterized *in vitro* T3SS function and *in vivo* pathogenicity of a number of strains encoding sited-directed point mutations in *yopD*. This resulted in the classification of mutants into three different classes based upon the severity of the phenotypic defects. To investigate the molecular and functional basis for these defects, we explored the effectiveness of RAW 264.7 cell line to respond to infection by representative YopD mutants of all three classes. Signature cytokine profiles could separate the different YopD mutants into distinct categories. The activation and suppression of certain cytokines that function as central innate immune response modulators correlated well with the ability of mutant bacteria to alter anti-phagocytosis and programmed cell death pathways. These analyses demonstrated that sub-optimal translocon pores impact the extent and magnitude of host cell responsiveness, and this limits the capacity of pathogenic *Yersinia* spp. to fortify against attack by both early and late arms of the host innate immune response.

## Introduction

The group of three human pathogenic *Yersinia* species are endowed with a Ysc-Yop type 3 secretion system (T3SS) that is encoded on a common 70 kb virulence plasmid [1]. This Ysc-Yop T3SS delivers into eukaryotic cells up to seven plasmid-encoded effector proteins – YopE, YopH, YopJ (also known as YopP), YopM, YopO (YpkA), YopT, and YopK [2–4], as well as a few uncharacterized chromosomal encoded proteins [5]. Most of the plasmid-encoded effectors have well-defined roles in immune modulation, including inhibition of phagocytosis, as well as hijacking proinflammatory and cell death signaling pathways. The net effect of these activities helps the pathogen to survive and thrive in the presence of a robust immune response during experimental infection of cellular and animal models [2–4,6–8].

Yop effector translocation requires the translocon apparatus assembled at the T3SS needle tip [9,10]. The Ysc-Yop T3SS translocon comprises three proteins – LcrV, YopB, and YopD. The hydrophilic LcrV protein is needle tip associated and probably facilitates assembly of YopB and YopD complexes [11–13]. The hydrophobic YopB and YopD proteins complex together and integrate into the host cell plasma membrane [14–18]. The prevailing idea is that Yop effector delivery occurs in one step through a uninterrupted needle-translocon pore conduit [19,20]. However, at some point, translocation occurs as a multistep process to enable the targeting of Yops after their secretion and association with the bacterial surface [21,22]. Despite knowledge of the individual bacterial components involved, a molecular understanding of the translocation mechanism is limited.

To bridge this knowledge gap, a number of studies have focused on revealing the function of YopD. Interestingly, YopD is both a translocator and a regulatory element controlling Yop synthesis in the bacterial cytoplasm [23–27]. The regulatory role is thought to manifest itself in the form of translation inhibition of Yop synthesis by direct binding to *yop* mRNA [26], association with the 30S ribosomal subunit [27], and hijacking of global RNA regulators [28]. These are important observations for mutants of YopD with impaired regulatory capacity overproduce Yops and fail to grow at high temperatures after just a few generations [23,29].

YopD mutant temperature sensitivity has thwarted studies aimed at elaboratoring the translocator role of this intriguing protein. This leads us to pry apart discrete functional domains of YopD by specifically disrupting by site-directed mutagenesis a helical domain present in the N-terminus [30] and in the C-terminus of YopD [31]. Significantly, recovered single substitution mutations resulted in YopD variants that maintained regulatory control over Yop synthesis, and the corresponding bacterial mutants were not growth restricted at high temperatures. Subsequent virulence testing in a mouse model of acute infection revealed three different attenuation phenotypes. Mutant bacteria producing YopD_I32K_ or YopD_I32P_ were only slightly attenuated when infected with low bacteria loads, but the infection was ultimately fatal [30]. Mutant bacteria producing YopD_A263P_ or YopD_A270P_ caused obvious signs of infection, but the infection was not fatal [31]. Mutant bacteria producing YopD_I262P_ or YopD_K267P_ were avirulent, with no visible signs of infection [31]. Complete attenuation arose from a non-functional translocon that was incapable of effector delivery, a phenotype indistinguishable from a *yopD* null mutant phenotype [31–33]. However, the other two mutant classes were still capable of effector translocation in classical tissue culture infection assays [30,31]. A summary of this phenotypic characterization is presented in Supplementary Table S1. The phenotypic differences may reflect important discrepancies in translocon conformation that consequently influence *in vivo* translocation efficiency. Hence, the aim of this study was to investigate this notion by exploring eukaryotic cell responsiveness to these phenotypic variants of YopD.

## Results

### Targeting focal complex proteins for inhibition of bacterial uptake

**Figure 1.**
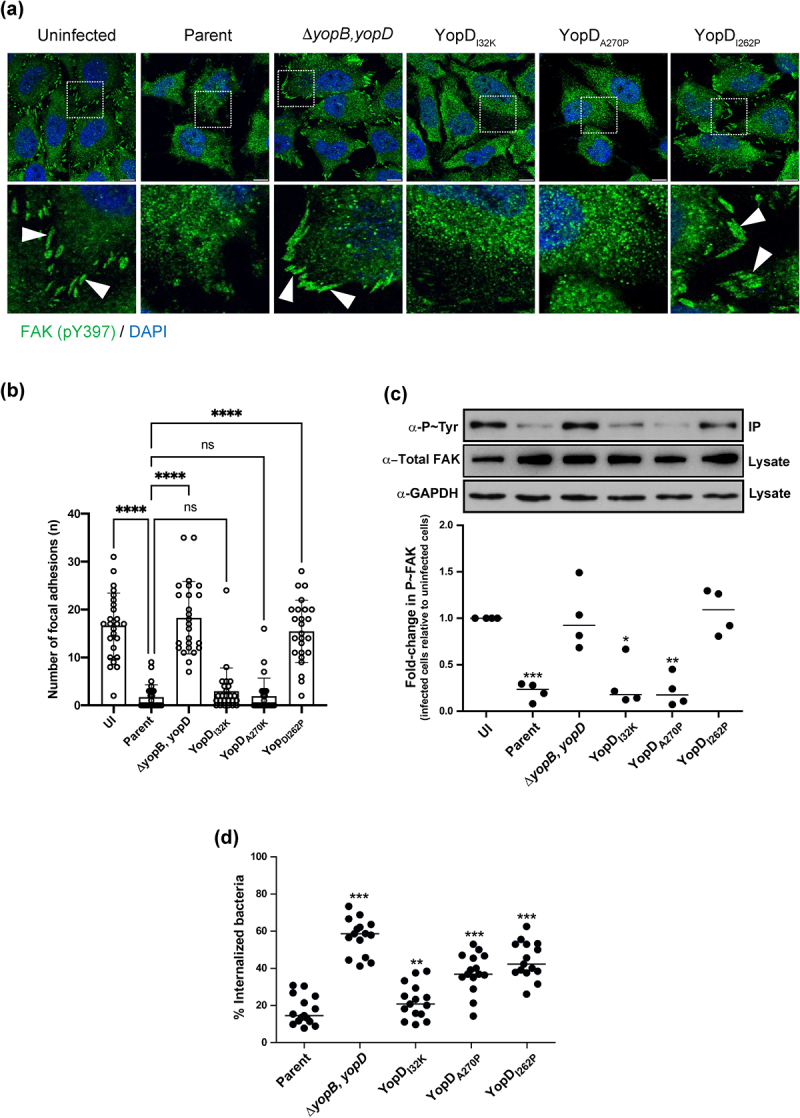
Anti-phagocytosis by *Y. pseudotuberculosis* is mediated through dephosphorylation of focal adhesion kinase. HeLa cells were infected with parental *Y. pseudotuberculosis* or strains producing the variants of YopD at a MOI of 20 (**a**) Representative confocal microscopy images of uninfected or infected HeLa cells with variants of *Y. pseudotuberculosis*. Arrowheads (white) shows FAK (pY397) stained focal adhesions (green). Nuclei were counterstained with DAPI (blue). Scale bars = 10 μm. **(b)** histogram indicates quantifications of focal adhesions per cell (*n* = 25 cells), shown in (**a**). Representative data from two independent experiments is presented. Significance was determined using one-way analysis of variance (ANOVA) with Dunnett’s post-test against the parent strain of *Y. pseudotuberculosis*. ****p < 0.0001, ***p < 0.001, **p < 0.01, *p < 0.05. Or ns = not significant. **(c)** at 10 min post infection, cells were lysed and FAK from each individual lysate was immunoprecipitated using anti-FAK antibodies bound to protein G-Sepharose. Equivalent amounts of eluted material was fractionated by SDS-PAGE, and following immunoblotting, membranes were incubated with anti-Phosphotyrosine antibody, clone 4G10, and then with HRP-conjugated anti-mouse antibodies. The displayed western blot image is one experiment. The scatter dot plot beneath this image represents the quantification achieved from four independent experiments (*n* = 4), and represents the fold change in recovered phosphorylated FAK. The results presented are the mean value of four independent experiments. The quantification was carried out using ImageJ software. The triple asterisk (***), double asterisk (**) and single asterisk (*) reflects the degree of significant difference with *p <* 0.001, *p <* 0.01, and *p <* 0.05, respectively, when compared to the uninfected control. In an a parallel set of experiments, RAW 264.7 cells were infected with parental *Y. pseudotuberculosis* or strains producing the variants of YopD at a MOI of 20 (**d**). At 30 min post infection, the cells were fixed with 4% PFA. Extracellular bacteria were stained using *Yersinia* antisera followed by Alexa568-conjugated antibody. The cells were then permeabilized with 0.5% Triton X-100 and both extra- and intracellular bacteria were stained with *Yersinia* antisera followed by Alexa488-conjugated antibody. Cells were counterstained using Hoechst. For each sample, the amount of total and extracellular bacteria were counted manually using a fluorescence microscope over three independent experiments (i.e. 5 fields of view per independent experiment). The percent internalised bacteria was calculated for each field of view (*n* = 15) and incorporated visualization of at least 150 eukaryotic cells per sample. The double asterisk (**) and single asterisk (*) reflect on the degree of significant difference with *p <* 0.01 and *p <* 0.0*5*, respectively, when compared to parental bacteria.

In the early stages of an infection, pathogenic *Yersinia* spp. establish close contact with the host cell via interactions with the host cell surface β1 integrin [34–36]. These interactions subsequently cause dephosphorylation of the focal complex protein, focal adhesion kinase (FAK), to trigger architectural rearrangements in the cytoskeleton and plasma membrane, which promotes phagocytosis by the eukaryotic cell [37]. To establish an extracellular replication niche, *Yersinia* utilize an active T3SS to deploy several early-acting Yop effectors to counteract the phagocytic response [8]. A specific example of this is the translocated YopH effector – a potent tyrosine phosphatase – that quickly dephosphorylates FAK to restrain the infected eukaryotic cells’ phagocytic characteristics [38]. Hence, we began by correlating the capacity of the minimally attenuated mutant YopD_I32K_, the modestly attenuated mutant YopD_A270P_, and the fully attenuated mutant YopD_I262P_, to translocate YopH (summarized in Supplementary Table S1 and references [30,31]) with the capability of translocated YopH to dephosphorylate FAK in HeLa cell monolayers. To accomplish this, HeLa cells infected with different variants of *Y. pseudotuberclosis* were stained with FAK (pY397) specific antibodies (Figure 1a,b). HeLa cells were chosen as a model system because of their popular usage in investigations of protein–protein interactions within the focal contact structure, which is abundant in these cells due to their flat and elongated morphology [38–40]. Compared to uninfected cells, we observed loss of focal adhesion staining in HeLa cells infected with the parent strain of *Y. pseudotuberculosis*. Similarly, there was significant loss of focal adhesions in cells infected with mutants producing either YopD_I32K_ or YopD_A270P_ (Figure 1a,b). Importantly, the Δ*yopB*, *yopD* double mutant and the mutant producing YopD_I262P_ both failed to cause perturbation of the focal adhesions (Figure 1a,b). This data was further confirmed by measurement of FAK phosphorylation levels in a population of immunoprecipitated FAK from infected cells (Figure 1c). Levels of phospho-FAK were considerably lower in HeLa cells exposed to parent bacteria or by those exposed to the mutants that produced the YopD_I32K_ or YopD_A270P_ variants (Figure 1c). In contrast, we observed high amounts of recovered phospho-FAK from the HeLa cells that were exposed to the Δ*yopB*, *yopD* double mutant and the mutant that produced the YopD_I262P_ variant (Figure 1c).

We then proceeded to investigate if these findings could be correlated to the inhibition of bacterial uptake (anti-phagocytosis). The RAW 264.7 macrophages were suitable for this purpose because the mechanism of *Y. pseudotuberculosis-*induced anti-phagocytosis is established in these professional phagocytic cells [41]. To address if *Y. pseudotuberculosis* can translocate YopE and YopH into RAW 264.7 macrophages, this cell line was infected with the parent *Y. pseudotuberculosis* strain and compared its effect with the strains containing different *yopD* mutations. This was followed by labeling the cells with the CCF2-AM FRET probe, and live cell imaging (Supplementary Figure S1a–e). The parent bacteria effectively translocated both YopE_86_-Bla and YopH_99_-Bla into the infected cells. We also observed the translocation of YopE_86_-Bla and YopH_99_-Bla in RAW 264.7 macrophages infected with the YopD_I32K_ or YopD_A270K_ mutants, although to a lesser extent than parental bacteria. In contrast, the YopD_I262P_ mutant was unable to translocate YopE_86_-Bla and YopH_99_-Bla into RAW 264.7 macrophages. These observations are in agreement to that reported for translocation of YopE and YopH into the HeLa epithelial cell line [31].

Next, we incubated the naïve RAW 264.7 cell monolayers with the various *Y. pseudotuberculosis* strains for 30 min, prior to processing them with a dual immunofluorescent staining procedure that was designed to distinguish between the internalized bacteria and those that remained extracellular. Significantly, both the parental bacteria and the mutant producing YopD_I32K_ could resist phagocytosis, with <20% of bacteria internalized (Figure 1d). In contrast, up to 60% of all cell-associated Δ*yopB*, *yopD* double mutant bacteria was internalized (Figure 1d). Interestingly, RAW 264.7 macrophages internalized around 40% of mutant bacteria producing either the YopD_I262P_ or the YopD_A270P_ variant (Figure 1d).

In total, these data indicate that the mutant producing YopD_I32K_ sufficiently targets Yop effectors, including YopE and YopH (Supplementary Figure S1 and Table S1) [30], and targeted effectors are fully functional inside the eukaryotic cell. Interestingly, despite the mutant producing YopD_A270P_ translocating YopE and YopH to levels similar to the mutant producing YopD_I32K_ (Supplementary Figure S1 and Table S1) [31], and also targeted YopH to focal complexes to dephosphorylate FAK, the YopD_A270P_ producing mutant failed to resist phagocytosis by macrophages. This indicates a disconnect between YopH effector delivery and function by the mutant producing YopD_A270P_ such that this YopD variant affects YopH activity after it is delivered to host cells. Finally, the mutant producing YopD_I262P_ is impaired in YopE and YopH translocation (Supplementary Figure S1 and Table S1) [31] and subsequently also in the measurable activities of internalized YopH.

### *Y. pseudotuberculosis* influences cell death signaling

**Figure 2.**
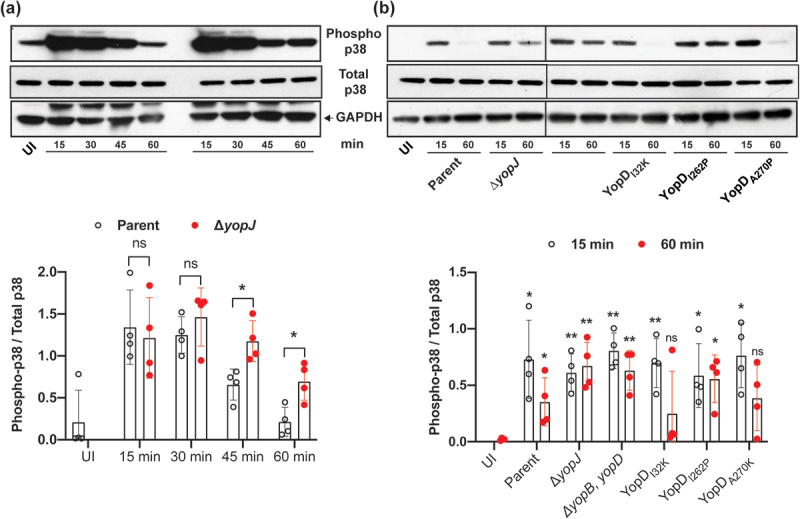
YopD variants control *Y. pseudotuberculosis* mediated suppression of p38 MAPK. RAW 264.7 macrophages were infected with parental *Y. pseudotuberculosis* or the Δ*yopJ* mutant strain at a MOI of 20 (**a**). At different time points post-infection (15, 30, 45, and 60 min), cells were lysed and equal amount of protein were resolved on SDS-PAGE. Active p38 was detected by western blot using phospho-p38 antibodies. Total p38 and GAPDH were used as a loading control. The displayed western blot image is one experiment. The scatter plot beneath this image represents the quantification achieved from four independent experiments (*n* = 4), and illustrates the time-dependent suppression of phospho-p38 by infection with parental bacteria compared to infection with the Δ*yopJ* mutant. In a subsequent series of experiments using the same conditions, RAW 264.7 macrophages were infected with parental *Y. pseudotuberculosis*, the Δ*yopJ* mutant strain, or strains producing variants of YopD (**b**). At two pre-selected time points post-infection (15 and 60 min), cells were lysed and equal amount of protein were resolved on SDS-PAGE. Active p38 was detected by western blot using phospho-p38 antibodies. Total p38 and GAPDH were used as a loading controls. The displayed western blot image is one experiment. The scatter dot plot beneath this image represents the quantification achieved from four independent experiments (*n* = 4), and illustrates the time-dependent suppression of phospho-p38 by infection with parental bacteria compared to infection with bacteria producing the various YopD mutants. In all cases, quantification of western blots was performed using ImageJ software. The phospho-p38 data was normalized against total p38. Significance was determined from biological replicates using a two-tailed, unpaired Student’s *t*-test. The double asterisk (**) and single asterisk (*) reflects the degree of significant difference with *p <* 0.01 and *p <* 0.05, respectively.

**Figure 3.**
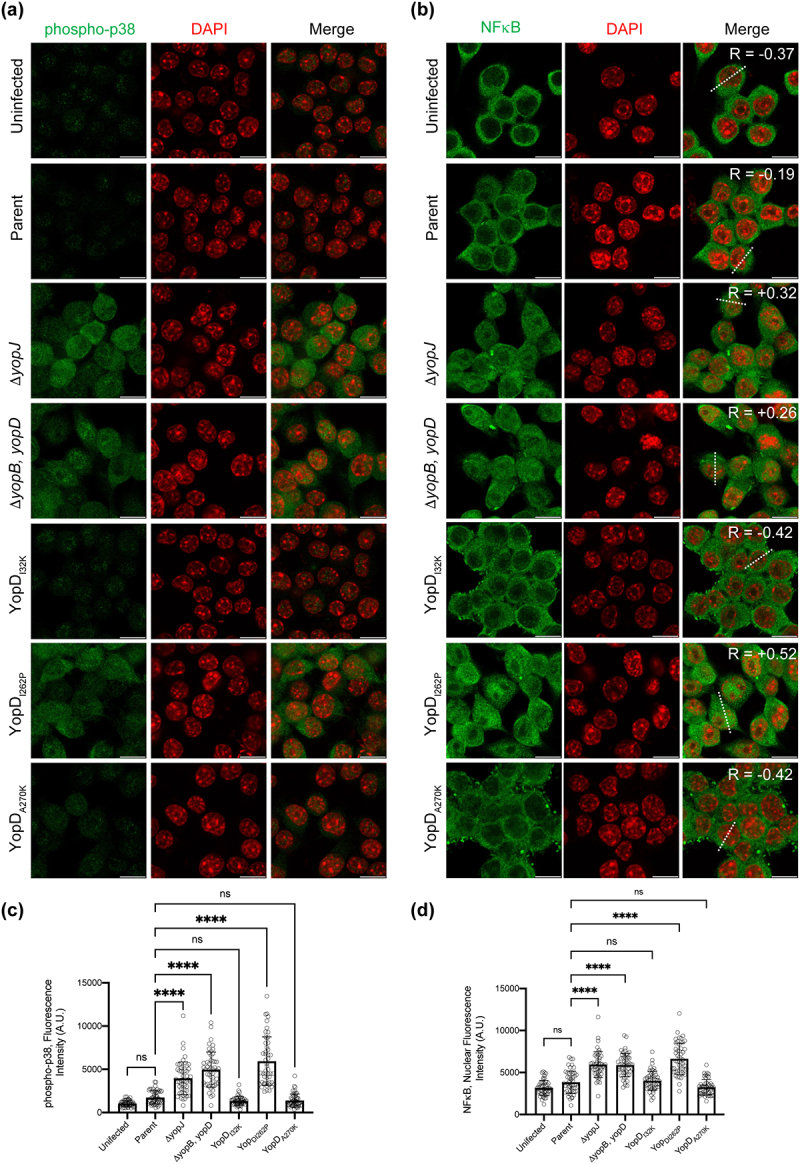
*Y. pseudotuberculosis*-mediated inhibition of p38 and NF-κB is influenced by the function of YopD. (**a-b**) RAW 264.7 cells were infected with parental *Y. pseudotuberculosis* or strains producing the variants of YopD at a MOI of 20. At 4 h post-infection, cells were fixed, permeabilized and incubated sequentially with anti phospho-p38 (**a**) or anti NF-κB (**b**) antibodies and then with AlexaFlour-488 or -568 conjugated secondary antibodies. Cells were counter-stained for nuclei with DAPI, and visualized by confocal microscopy. The dashed lines in the panel furtherest to the right in part (**b**) were used for calculation of Pearson coefficient for colocalization of NF-κB and DAPI. (**c**) The histogram to the left indicates quantification of phospho-p38 staining intensity of the uninfected and infected RAW 264.7 cells. (**d**) The histogram to the right indicates quantification of nuclear NF-κB staining intensity of the uninfected and infected RAW 264.7 cells. The quantification was carried out using ImageJ software. Data points indicate fluorescence intensity for randomly selected cells from two independent experiments (*n* = 50 cells). Histogram show mean ± S.D. Significance was determined from replicates using a one-way analysis of variance (ANOVA) with Dunnett’s post-test against cells infected with parent strain of *Y. pseudotuberculosis*. ****p < 0.0001, *p < 0.05. Or ns = not significant. Scale bars = 20 μm.

Cell death signaling cascades involving mitogen‐activated protein (MAP) kinases are triggered by close contact between pathogenic *Yersinia* spp. and the target host cell, and this ultimately determines the viability of the infected cell [42–45]. Further, it is the late targeting by bacteria of Yop effectors, such as YopJ, which primarily influence these signaling events [42–48]. Hence, we investigated mutant bacteria producing either YopD_I32K_, YopD_I262P_, or YopD_A270P_ for the ability to carry-out YopJ-dependent influences on intracellular signaling events within infected eukaryotic cells. This was done by first examining the status of MAPK pathway activation by detecting the levels of MAP kinase p38 phosphorylation in *Y. pseudotuberculosis*-infected RAW 264.7 macrophages. We initially performed a time-course experiment to compare the degree of p38 activation in naïve RAW264.7 macrophages infected with parent bacteria and the Δ*yopJ* deletion mutant. This mutant was used because translocated YopJ suppresses phosphorylation of p38, which, as a consequence, decreases NF-κB production [42–48]. In our experimental setup, we observed that the phospho-p38 levels fell below the basal levels we had observed in uninfected cells after 60 min post-infection with the parental bacteria (Figure 2a). In contrast, the macrophages that were infected with the Δ*yopJ* deletion mutant sustained higher phospho-p38 levels (Figure 2a). Next, we examined the activation of p38 in RAW 264.7 macrophages that were infected with the different *Y. pseudotuberculosis* YopD point mutants. Significantly, at 60 min post-infection the macrophages with the fully attenuated mutant YopD_I262P_ sustained high phospho-p38 levels equivalent to those infected with the Δ*yopJ* deletion mutant or the Δ*yopB*, *yopD* double mutant that lacked the translocon (Figure 2b). Simultaneously however, the macrophages that were infected with the mutants that produced the YopD_I32K_ or YopD_A270P_ variants had diminished phospho-p38 levels (Figure 2b). We performed immunofluorescent microscopy with phospho-p38 antibodies on infected macrophages to corroborate this data. The quantification of fluorescence intensity confirmed that YopD_I32K_ or YopD_A270P_ variants had diminished phospho-p38 levels, while the YopD_I262P_ variant sustained high phospho-p38 levels (Figure 3a,c).

Additionally, we examined the nuclear NF-κB levels in the infected RAW 264.7 macrophages by quantifying the signal derived from the NF-κB p65 fluorescent antibody to determine if the data coincided with the activation status of NF-κB. Compared to uninfected cells, all *Y. pseudotuberculosis-*infected RAW 264.7 macrophages accumulated a large pool of NF-κB in their nuclei (Figure 3b,d). Moreover, a clear tendency was established in regard to the NF-κB suppression in the macrophages that were infected with bacteria that possessed an active T3SS (i.e. parental bacteria and the mutants producing the YopD_I32K_ or YopD_A270P_ variants) (Figure 3b,d). In contrast, the median values of NF-κB were greater in the mutants that lacked a functional T3SS translocon (i.e. the Δ*yopB*, *yopD* double mutant and the mutant producing YopD_I262P_) or those that lacked the YopJ effector (i.e. the Δ*yopJ* deletion mutant) (Figure 3b,d). Importantly, there was a positive correlation for NF-κB and DAPI staining in cells infected with variants of *Y. pseudotuberculosis* that lacked the active T3SS or Δ*yopJ* deletion mutant, while negative correlation was observed for the cells that were infected with variants of *Y. pseudotuberculosis* that had active T3SS (Figure 3b,d).

**Figure 4.**
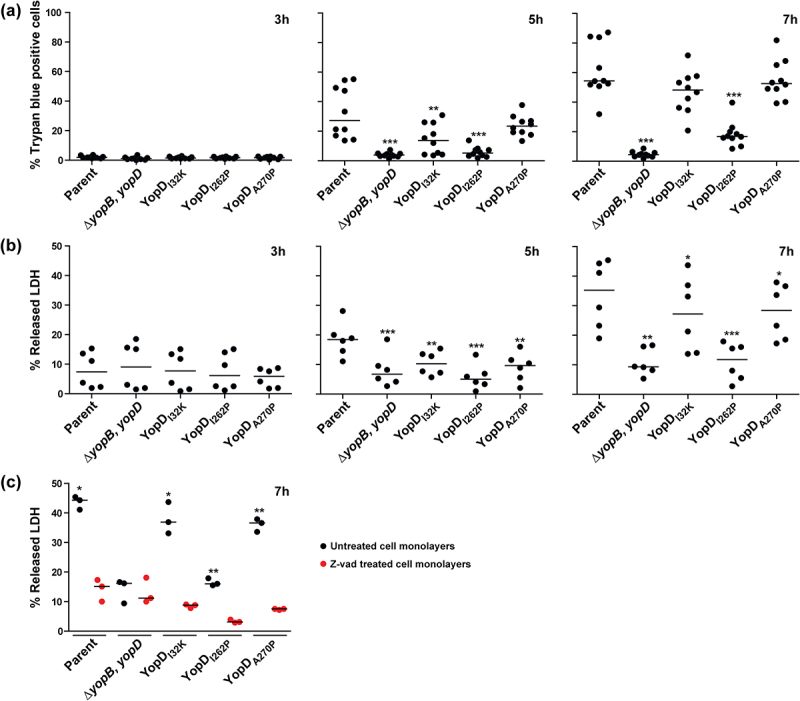
Kinetics of RAW 264.7 macrophage cell death induced by *Y. pseudotuberculosis*. RAW 264.7 macrophages were infected with parental *Y. pseudotuberculosis* or strains producing variants of YopD at a MOI of 20 at different time points. Cell death was quantified by the Trypan blue exclusion assay (**a**) or the LDH release assay (**b**). For the Trypan blue exclusion assay, cells were microscopically evaluated after being overlaid with 2% Trypan blue (*n* ≥ 500 cells in a total of 10 microscopic field fields of view). For the LDH release assay, 50 μl of cell supernatants were transferred to a flat bottom 96-well ELISA plate. A 50 μl reaction mix was added to each well and plate was incubated at room temperature in the dark for 30 min then LDH released from the cells was evaluated using a TECAN plate reader (*n* = 6). The Δ*yopB*, *yopD* null mutant, and the YopD_I262P_ point mutant were less able to induce cell death when compared to all other strains including parental bacteria. The pan-caspase inhibitor, z-VAD reduced LDH release from macrophage cells exposed to parental bacteria or the YopD_I32K_ or YopD_A270P_ point mutants (**c**). In this case, cell monolayers were pre-incubated with 25 μM z-VAD for 2 h and then infected with bacteria at a MOI of 20. After 7 h post-infection, the extent of LDH released assessed (*n* = 3). Significance was determined from biological replicates using a two-tailed, unpaired Student’s *t*-test. The triple asterisk (***), double asterisk (**), and single asterisk (*) reflect the degree of significant difference with *p* < 0.001, *p* < 0.01, and *p* < 0.05, respectively.

Given that activation of the MAPK pathway and NF-κB signaling causes cell death, we proceeded to investigate whether the infection of RAW 264.7 macrophages that were infected with different *Y. pseudotuberculosis* point mutants caused proportional differences in the extent of macrophage cell death. We used the Trypan blue dye exclusion assay to track the fate of the infected RAW 264.7 macrophages up to 7 h post-infection. We observed a gradual accumulation of dead macrophage cells from 5 h post-infection by all *Y. pseudotuberculosis* strains, with the exception of the Δ*yopB*, *yopD* double mutant (Figure 4a, middle panel). At 7 h post-infection, a median range between 50% and 55% of macrophage cells that were infected with parental bacteria, or the strains producing either YopD_I32K_ or YopD_A270P_ had died (Figure 4a, right panel). The extent of the cell death we observed far exceeded the average percent of dead cells (<20%) that followed an infection with the YopD_I262P_-producing strain (Figure 4a, right panel). In contrast, the vast majority of cells within the monolayer that were infected with the Δ*yopB*, *yopD* double mutant remained viable 7 h post-infection (Figure 4a, right panel). This data was independently confirmed with the use of a LDH release assay (Figure 4b). The extent of LDH release into the culture media from the macrophage cells that were infected with parental bacteria, or the strains producing either YopD_I32K_ or YopD_A270P_, far exceeded the LDH released from cells that were infected with the Δ*yopB*, *yopD* double mutant or the strain that produced YopD_I262P_ (Figure 4b). Hence, RAW 264.7 macrophage cells undergo cell death when infected with parental bacteria, or the strains producing either YopD_I32K_ or YopD_A270P_, and to a significantly lesser extent with the strain producing YopD_I262P_, but not with the Δ*yopB*, *yopD* double mutant that lack the translocon. It is likely that this cell death is caspase dependent, because RAW 264.7 macrophage monolayers pre-treated with the pan-caspase inhibitor Z-Val-Ala-Asp fluoromethyl ketone (z-VAD-FMK) prior to infection resulted in a marked reduction in the amount of LDH released compared to untreated infected cells (Figure 4c).

In total, these data indicate that the mutant that produced YopD_I262P_ cannot translocate late effectors, including YopJ, into the infected cells, and that this causes negative repercussions in the signaling of cell death. On the other hand, the mutants that produced either YopD_I32K_ or YopD_A270P_ maintained an ability to translocate late Yop effectors into infected cells. Moreover, the YopJ translocated by these two mutants is functional.

### Cytokine profiles generated from *Y. pseudotuberculosis*-infected RAW 264.7 macrophages

**Figure 5.**
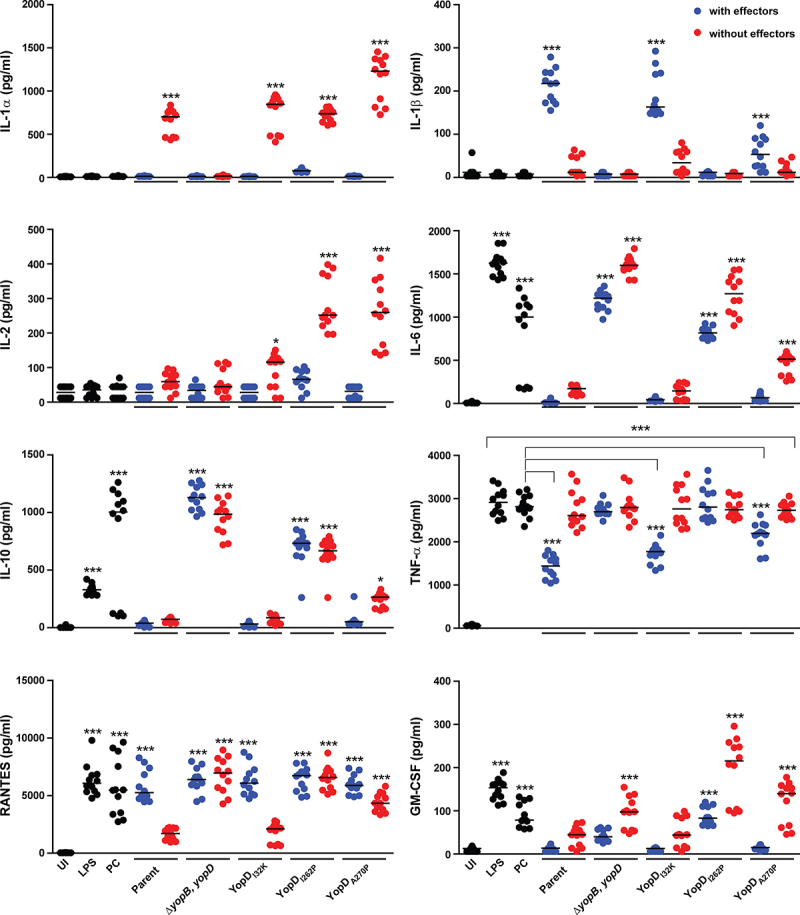
Macrophage cytokine production profiles that are able to distinguish between mutant strains of *Y. pseudotuberculosis*. RAW 264.7 cells were infected with different strains of *Y. pseudotuberculosis* at a MOI of 20. Cleared culture supernatants were collected after 4 h post infection, then quantified for cytokine production using the Q-Plex mouse cytokine-Screen (16-plex). Differential cytokine expression profiles in response to stimulation conditions are clearly visible using a scatter plot representation, with each circle representing an independent data point and the mean value shown as a horizontal line (*n* = 12). Black circles are primary control conditions represented by uninfected cells (UI), and cells exposed to either 1 µg/ml purified *E. coli* lipopolysaccharide (LPS) or to *Y. pseudotuberculosis* YPIII that has been cured of the virulence plasmid encoding for the Ysc-Yop T3SS (PC). Blue circles are cells exposed to parental *Y. pseudotuberculosis* or strains producing variants of YopD that still contain a full repertoire of immune-modulatory Yop effectors. Red circles are cells exposed to isogenic parental *Y. pseudotuberculosis* or strains producing variants of YopD that are devoid of all plasmid-encoded immune-modulatory Yop effectors (designated as multiple *yop* mutants – MYM). Significance was determined from biological replicates using a two-tailed, unpaired Student’s *t*-test. The triple asterisk (***), double asterisk (**), and single asterisk (*) reflect the degree of significant difference with *p* < 0.001, *p* < 0.01, and *p* < 0.05, respectively. For the most part, the significance asterisks are relative to the untreated cells.

We have documented different phenotypes of the YopD point mutants interacting with eukaryotic cells on the basis of measured activity of internalized YopH (early translocated effector – anti-phagocytosis activity) and internalized YopJ (late translocated effector – host cell death) (summarized in Supplementary Table S1). However, it is recognized that innate immune signaling of the host is also influenced by the direct recognition of the YopB/YopD translocon proteins [33,49–53]. In light of this, we investigated if it was possible to distinguish the *Y. pseudotuberculosis* YopD point mutants by their modulation of an array of cytokines produced by the infected RAW 264.7 macrophages. For convenience, we used the Mouse Cytokine-Screen 16-plex analysis (tebu-bio SAS) that profiled 16 cytokines (IL-1α, IL-1β, IL-2, IL-3, IL-4, IL-5, IL-6, IL-10, IL-12p70, IL-17, MCP-1, IFNγ, TNFα, MIP-1α, GM-CSF, and RANTES). To validate the experimental approach, we compared the cytokine levels in the cell-free supernatants from the naïve macrophage monolayers that had remained untreated and from the naïve cells that had been exposed to either purified bacterial LPS or to *Y. pseudotuberculosis* bacteria that had been cured of the virulence plasmid that encodes for the Ysc-Yop T3SS. The raw data concerning the individual cytokines are presented as a scatter dot plot in Figure 5, for production profiles that are able to distinguish between mutant strains of *Y. pseudotuberculosis*, and Supplementary Figure S2, for production profiles with no power to discriminate between mutant strains of *Y. pseudotuberculosis*. Principal component analysis (PCA) was used to model the cytokine profile in the individual control samples (i.e. 12 replicate samples for each control). This defined a clear normality boundary without sample outliers, which indicated that a high degree of reproducibility was achieved among the replicated samples (Supplementary Figure S3a). As expected, cytokine production that resulted as a consequence from exposure to LPS or to the plasmid cured strain clustered together and was clearly separated from the cytokine production profile obtained from uninfected cells (Suplementary Figure S3a). We concluded that the host cell cytokine responsiveness upon exposure to LPS or the plasmid-cured strain was essentially the same, and which is utterly distinct from that produced by untreated host cells.

A concern with using the macrophage-like RAW264.7 cell line to measure immune responses is that this cell line dramatically differs from primary macrophages. Hence, to validate these cytokines screened using RAW264.7 cells, we also isolated peripheral blood mononuclear cells (PBMCs) from human blood and infected them with different isogenic variants of *Y. pseudotuberculosis*. Consistent with the results obtained for RAW264.7 cells, we observed a similar TNFα production profile in PBMCs (Supplementary Figure S4). Thus, while some differences may be missed in the cytokine response to *Y. pseudotuberculosis* infection of RAW264.7 cells, our data suggest comparable response patterns between RAW264.7 cells and primary cells. We proceeded to analyze cytokine production from naïve RAW 264.7 macrophages that had been exposed to five different *Y. pseudotuberculosis* variants – the parental bacteria with a fully functional T3SS, the Δ*yopB*, *yopD* null mutant that lacked both hydrophobic translocon components, and the three point mutants that produced YopD_I32K_, YopD_A270P_, and YopD_I262P_, respectively. To dissect these profile patterns without bias, we treated each sample replicate individually and used PCA to compare all 12 replicates with the LPS and plasmid cured control samples first analyzed in Supplementary Figure S3a for each of the five strains. This revealed that host cells that were exposed to the pore deficient translocator mutant that lacked both YopB and YopD produced a minimalistic cytokine production profile that was essentially indistinguishable from the control profiles (Suplementary Figure S3b). In contrast, the cytokine production that arose in response to the parental bacteria and to the YopD_I32K_ mutant deviated substantially from the normality boundary and this was demarcated by responsiveness to LPS, plasmid cured and the Δ*yopB*, *yopD* null mutant (Supplementary Figure S3b). Although to a lesser extent, deviation was also evident in the responses to both YopD_A270P_ and YopD_I262P_, with the former trending toward responses to the parental bacteria and the YopD_I32K_ mutant, while the latter trended in its own distinct cluster (Supplementary Figure S3b).

**Figure 6.**
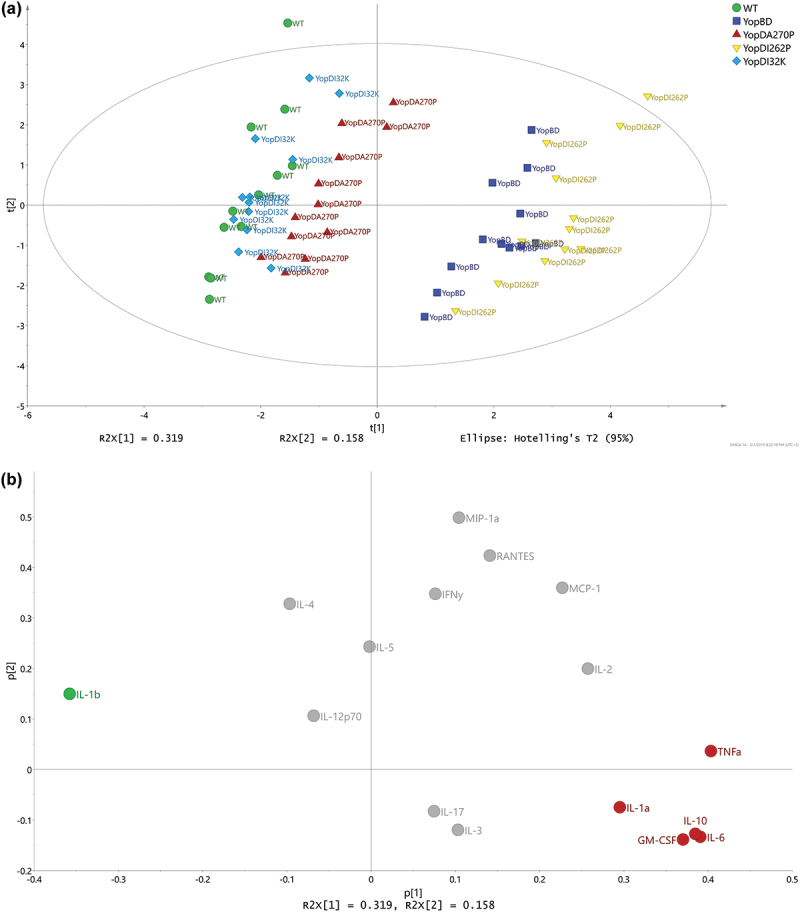
Multivariate analysis of cytokine profiles from RAW 264.7 cells exposed to effector positive *Y. pseudotuberculosis*. (**a**) Scores (t1/t2) plot for the PCA model (*N* = 60, *K* = 16, 2 comp., R2X(cum) = 0.477, Q2(cum) = 0.229) fitted for the five strains WT (YPIII/pIB102), YopBD (YPIII/pIB619 lacking *yopB* and *yopD*), YopD_I32K_ (YPIII/pIB60501 with *yopD* containing the codon substitution I32K), YopD_I262P_ (YPIII/pIB63301 with *yopD* containing the codon substitution I262P), and YopD_A270P_ (YPIII/pIB63304 with *yopD* containing the codon substitution A270P). It was observed that the main structure in data differentiated the WT, YopD_I32K_, and YopD_A270P_ samples from the YopBD and YopD_I262P_ samples. (**b**) The corresponding loadings (p1/p2) plot for the PCA model in Figure 5. The cytokines ending up with a common covariance pattern were colored referring to the positions in the loadings plot. It was observed that IL-6, IL-10, and GM-CSF (red coloring) were highly correlated and could be linked to the observed groupings in Figure 5, indicating in higher concentrations for both YopDB and YopD_I262P_. Also, IL-1β (green coloring) indicated to separate the two observed groups based upon higher concentrations for WT, YopD_I32K_, and YopD_A270P_.

Next, we performed a PCA to model the replicate samples of the five key strains to permit better resolution and reveal distinctive pattern profiles. We identified two distinct groups. The first group consisted of samples from RAW 264.7 macrophages that had been exposed to the YopD_I32K_- or YopD_A270P_-producing strains or parental bacteria (Figure 6a). The second group consisted of samples from RAW 264.7 macrophages that had been exposed to the YopD_I262P_-producing strain or the translocon deficient Δ*yopB*, *yopD* double mutant (Figure 6a). The cytokines IL-1α, IL-6, IL-10, TNF-α, and GM-CSF (Figure 6b, red color coding) were positively associated to demarcating the group composed of the YopD_I262P_-producing strain and the translocon deficient Δ*yopB, yopD* double mutant. In contrast, the cytokine IL-1β (Figure 6b, green color coding) was positively associated to demarcating the group composed of the YopD_I32K_- or YopD_A270P_-producing strains or parental bacteria. This modeling corroborates the raw data for this group of cytokines (Figure 5). The model predicted that the remaining cytokine profiles would not be linked to the separation of the two distinctive groups. This is entirely consistent with these cytokine profiles without significant differences among the distinct *Yersinia* strains (Suplementary Figure S2). It also corroborates that some cytokines, such as IFNγ, was a product of immune cells other than macrophages. Hence, a subset of cytokine production data that was collected from the RAW 264.7 macrophage infection model could demarcate genetically defined *yopD* mutants.

### Cytokine production by RAW 264.7 macrophages caused by Yop effector recognition

Host innate immune signaling can be potentiated by the recognition of the translocated Yop effectors [42–48,54–58]. Hence, we pondered over the host cell response to Yop effectors translocated by parental bacteria, the Δ*yopB*, *yopD* double mutant, and the three YopD point mutant backgrounds. To facilitate the required analyses, we generated a new cytokine production profile to these five strains in which we established a multiple *yop* mutant (MYM) via a sequential removal of all known plasmid encoded translocated Yop effectors – namely YopH, YopM, YopE, YopK, YpKA, and YopJ. Scatter dot plots display the raw cytokine levels in the 12 sample replicates of cleared supernatants from RAW 264.7 macrophages that had been exposed to all five *Y. pseudotuberculosis* strains that now also lack the six effectors (Figure 5 and Supplementary Figure S2). Again, PCA was used to juxtapose these cytokine profiles with those recovered from the cells that had been exposed to either purified bacterial LPS or *Y. pseudotuberculosis* bacteria cured of the virulence plasmid. Host cells that had been exposed to the pore deficient translocator mutant that lacked both YopB and YopD as well as all Yop effectors (Δ*yopB*, *yopD*-MYM) produced a minimalistic cytokine production profile that was essentially indistinguishable from the control profiles generated by exposure to LPS or the plasmid cured strain (Supplementary Figure S3c). In contrast, cytokine production in response to effector minus (MYM) bacteria that possessed a fully functional T3SS translocon (WT-MYM) or had defects defined by point mutations in the YopD translocator – YopD_I32K_-MYM, YopD_A270P_-MYM, and YopD_I262P_-MYM – fell well outside of the normality boundary demarcated by responsiveness to LPS, plasmid cured and the Δ*yopB*, *yopD*-MYM mutant (Supplementary Figure S3c).

**Figure 7.**
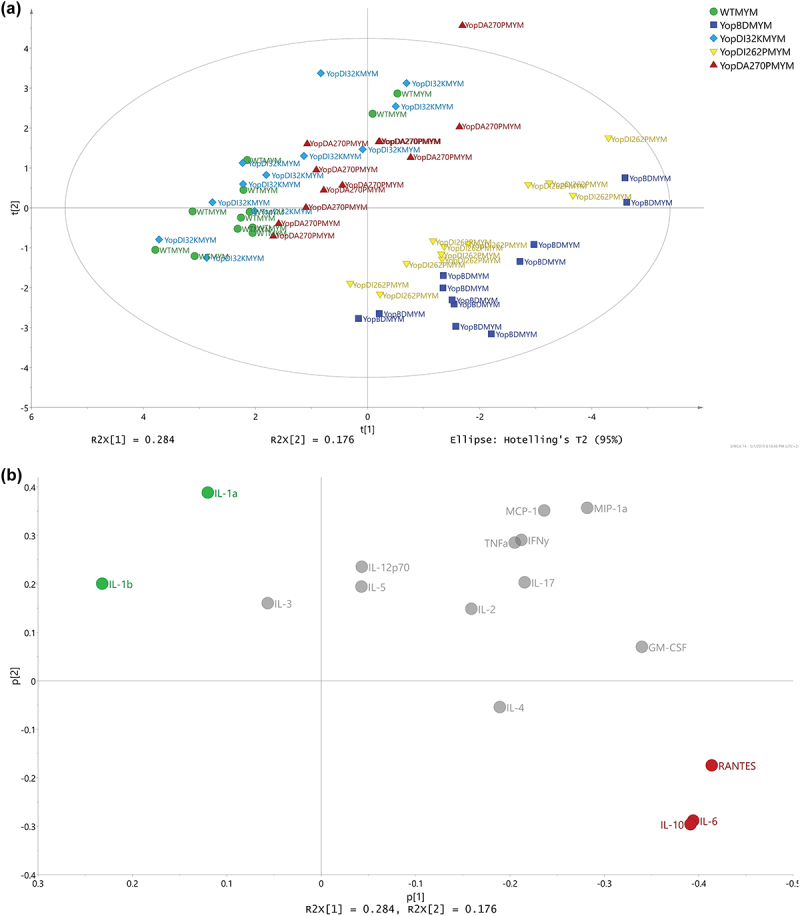
Multivariate analysis of cytokine profiles from RAW 264.7 cells exposed to *Y. pseudotuberculosis* strains lacking the translocated effectors. (**a**) Scores (t1/t2) plot for the PCA model (*N* = 60, *K* = 16, 2 comp., UV-scaled, R2X(cum) = 0.459, Q2(cum) = 0.134) fitted for the five multiple *yop* mutant (MYM – lacking *yopH, yopM, yopE, yopK, yopJ,* and *ypkA*) strains – WTMYM (YPIII/pIB29MEKJA – parent), YopBDMYM (YPIII/pIB29MEKJABD – MYM also lacking *yopB* and *yopD*), YopD_I32K_MYM (YPIII/pIB29MEKJA60501 – MYM with *yopD* containing the codon substitution I32K), YopD_I262P_MYM (YPIII/pIB29MEKJA63301 – MYM with *yopD* containing the codon substitution I262P), and YopD_A270P_MYM (YPIII/pIB29MEKJA63304 – MYM with *yopD* containing the codon substitution A270P). It was observed that the main structure in data differentiated the WTMYM, YopD_I32K_MYM, and YopD_A270P_MYM samples from the YopBDMYM and YopD_I262P_MYM samples. (**b**) The corresponding loadings (p1/p2) plot for the PCA model in Figure 6a. The cytokines ending up with a common covariance pattern were colored referring to the positions in the loadings plot. It was observed that IL-6, IL-10, and RANTES (red coloring) were highly correlated and could be linked to the observed groupings in Figure 6a, indicating in higher concentrations for both YopBDMYM and YopD_I262P_MYM. Also, IL-1α and IL-1β (green coloring) indicated to separate the two observed groups based upon higher concentrations for WTMYM, YopD_I32K_MYM, and YopD_A270P_MYM.

As these analyses indicated that the Δ*yopB*, *yopD*-MYM mutant could serve as a baseline response control, we performed a PCA to model the replicate samples of the five MYM strains. Two distinctive pattern profiles were highlighted – the first consisted of samples from RAW 264.7 macrophages that had been exposed to WT-MYM, YopD_I32K_-MYM, or YopD_A270P_-MYM and the second consisted of samples exposed to YopD_I262P_-MYM and Δ*yopB*, *yopD*-MYM mutant (Figure 7a). This was similar to what we had observed in the strains with the effectors (compare Figure 7a with 6a). The cytokines IL-6, IL-10, and RANTES (Figure 7b, red color coding) were positively associated to demarcating the group composed of YopD_I262P_-MYM and Δ*yopB*, *yopD*-MYM mutant. In contrast, the cytokines IL-1α and IL-1β (Figure 7b, green color coding) was positively associated to demarcating the group composed of the YopD_I32K_-MYM, YopD_A270P_-MYM, and WT-MYM.

In summary, the strains indicated similar cytokine profile trends regardless of being in the effector positive or effector negative backgrounds (compare Figure 7a with 6a). However, different specific cytokine response patterns were identified to account for the demarcation of two distinctive groups within these two respective backgrounds (compare Figure 7b with Figure 6b) This reflects an ability of both the translocon pore and the translocated effectors to modulate cytokine production.

### Translocon pore recognition specifically modulates production of a subset of cytokines

**Figure 8.**
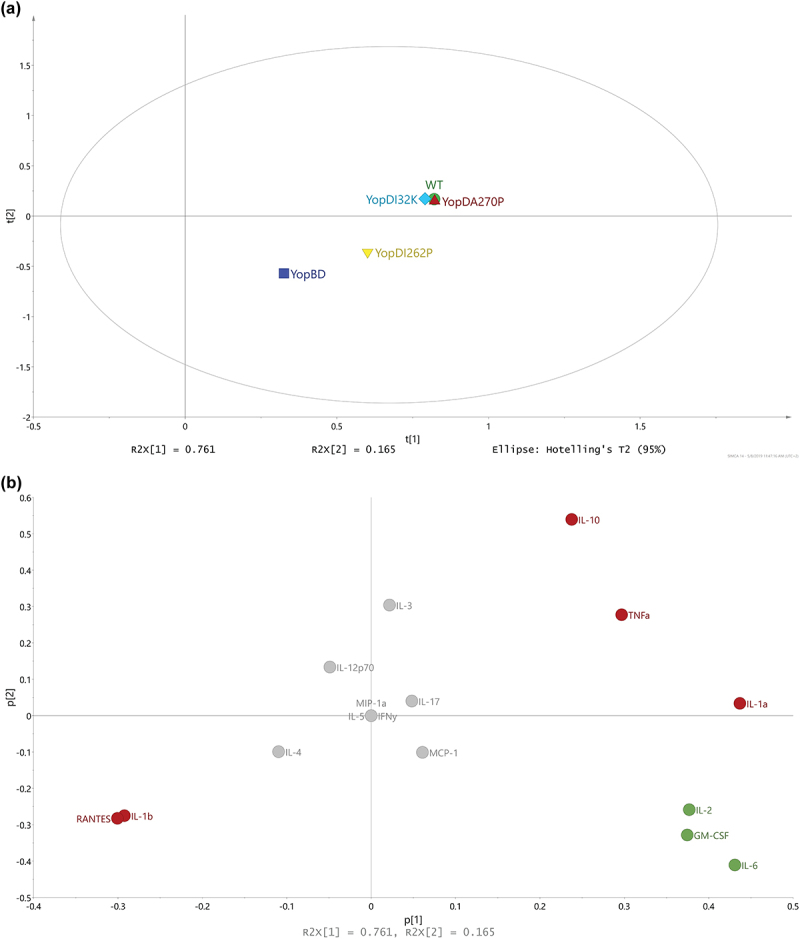
Using multivariate analysis to compare cytokine profiles from RAW 264.7 cells exposed to *Yersinia* strains with or without the translocated effectors. (**a**) Scores (t1/t2) plot for the PCA model (*N* = 5, *K *= 16, 2 comp., non-treatment, R2X(cum) = 0.926, Q2(cum) = 0.695) fitted for the effect profiles generated by OPLS-DA modelling, one model per strain against its corresponding multiple *yop* mutant (MYM). For each strain, an OPLS-DA model was fitted in order to separate the two treatments (i.e. strains with and without the Yop effectors) and the resulting predictive loadings (pp) represented the overall effect between the two treatments. It was obvious that the WT, YopD_I32K_, and YopD_A270P_ strains presented with a highly similar effect respective to the presence or absence of the Yop effectors. It was also apparent that the effect for YopD_I262P_ relative to the presence and absence of Yop effectors was neither similar to the YopBD strain nor similar to the other three strains. (**b**) The corresponding loadings (p1/p2) plot for the PCA model shown in Figure 7a. The coloring of cytokines was made corresponding to the trends in effects due to the presence and absence of Yop effectors. It was observed that IL-1α, IL-1β, IL-10, RANTES, and TNFα (red coloring) were clearly linked to similarities between the WT, YopD_I32K_, and YopD_A270P_ strains, relative to the YopBD and YopD_I262P_ strains. Also, due to the orthogonal directions in the plot, it was observed that IL-2, IL-6, and GM-CSF (green coloring) indicated that the highly similar WT, YopD_I32K_, and YopD_A270P_ strains were not identical.

After we characterized cytokine production from RAW 264.7 macrophages that had been exposed to translocator mutants in the presence of effectors (see Figure 6) and in the absence of effectors (see Figure 7), we intended to pry apart this information to distinguish relative contributions of the (1) translocon pore and (2) effector translocation to the cytokine production by infected host cells. In this analysis, we performed a pairwise comparison of the cytokine response profiles generated by two isogenic strains that only differed by the presence or absence of the Yop effectors. A PCA model was fitted with regard to the OPLS-DA effect profiles to investigate whether the magnitude of the cytokine responses differed between the strains (Figure 8a). This revealed that the parent and the YopD_I32K_ or YopD_A270P_ producing bacteria clustered together, which implies that the host cells reacted similarly to these three strains irrespective of the presence or absence of effectors (Figure 8a). In contrast, we observed a distinctive separation between the Δ*yopB*, *yopD* mutant background and the YopD_I262P_ mutant background (Figure 8a). The basis for this separation reflects that either YopB or certain Yop effectors that were produced by the YopD_I262P_ mutant could still modulate aspects of innate immune recognition despite the mutant being impaired for early and late effector targeting (see Figures 1–4, and Supplementary Table S1).

To investigate what cytokines were affected, we consulted the loadings (Figure 8b). These indicated a clear grouping of certain cytokine production profiles, which highlighted a similar discretionary power for some cytokines. The most prominent examples of this are the cytokines IL-1β and RANTES (positive associations between the presence and absence of effectors) and the cytokines IL-1α, IL-10, and TNF-α (negative associations between the presence and absence of effectors) (Figure 8b, red color coding). These particular cytokine responses are likely responsible for why parent and the YopD_I32K_ or YopD_A270P_ producing bacteria cluster together. We also identified IL-2, IL-6, and GM-CSF responses (Figure 8b, green color coding) that might explain the phenotypic differences that subtly distinguish the parent from the YopD_I32K_ or YopD_A270P_ producing bacteria (see Figures 1–4, and Supplementary Table S1).

**Figure 9.**
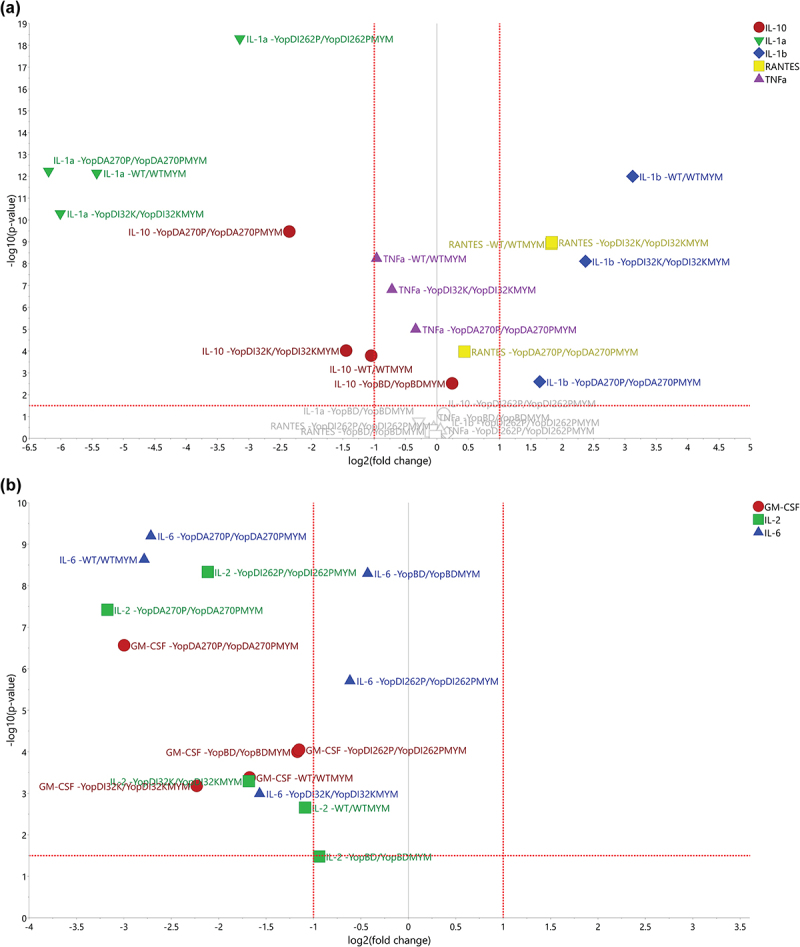
Using volcano plots to compare the fold-change of specific cytokine responses to bacteria with or without effectors). The volcano plots were generated for the distinct treatment effects colored red (**a**) and green (**b**) as visualized in Figure 8b. The y-axis refers to the independent *t*-tests (*p*-values) and the threshold was set at the 5% significance level (*α *= 0.05), referring to the treatment effects for each strain and cytokine. The x-axis refers to the corresponding fold change values (effect of the presence of Yop effectors divided by the effect of the absence of Yop effectors). (**a**) It was obvious that the strains WT, YopD_I32K_, and YopD_A270P_ indicated clear Yop effector-dependent treatment effects for IL-1α, IL-1β, IL-10, RANTES, and TNFα. It was also noticeable that IL-1α, IL-10, and TNFα ended up with negative fold changes, i.e. a boost in the particular cytokine response due to the loss of Yop effectors, while IL-1β and RANTES ended up with positive fold changes due to the loss of Yop effectors lessening the particular cytokine response. The strain YopD_I262P_ indicated a clear treatment effect only for IL-1α, while the strain YopBD indicated only a small treatment effect for IL-10. In contrast, in (**b**) we observe that the cytokines IL-2, IL-6, and GM-CSF could distinguish between the effector positive and effector negative strains within the WT, YopD_I32K_, and YopD_A270P_ backgrounds. In particular, IL-2 and GM-CSF distinguishes the YopD_A270P_ background from WT and YopD_I32K_, whereas IL-6 separates the YopD_I32K_ background from WT and YopD_A270P_.

To investigate these possibilities, we generated volcano plots that would allow us to compare the fold-change of specific cytokine responses (i.e. to the presence of effectors versus the absence of effectors) with a statistical significance analysis with the use of the Student's *t*-test. This test revealed that the extent of fold change for the cytokines IL-1α, IL-1β, IL-10, RANTES, and TNF-α were indeed similar for parent versus parent-MYM, YopD_I32K_ versus YopD_I32K_-MYM, and YopD_A270P_ versus YopD_A270P_-MYM (Figure 9a). Hence, this explains why these bacteria cluster together (see Figure 8a). A similar fold change of cytokine IL-1β between the parent versus WT-MYM, the YopD_I32K_ mutant versus YopD_I32K_-MYM, and the YopD_A270P_ mutant versus YopD_A270P_-MYM was observed, whereas no significant difference in fold change was observed for the Δ*yopB*, *yopD* mutant backgrounds or the YopD_I262P_ mutant backgrounds (Figure 9a). Moreover, the fold change of IL-1α between YopD_I262P_ versus YopD_I262P_-MYM differed significantly from the parent, YopD_I32K_ and the YopD_A270P_ bacterial cluster, and also from the Δ*yopB*, *yopD* mutant background where we did not observe any significant difference in fold change (Figure 9a). In addition, the fold change of IL-10 between the Δ*yopB*, *yopD* mutant and the Δ*yopB*, *yopD*-MYM mutant differed significantly from that observed for the parent and the YopD_I32K_ or YopD_A270P_ backgrounds, and from the YopD_I262P_ mutant background (Figure 9a). Clearly therefore, IL-1α, IL-1β, and IL-10 play a major part in discriminating the parent, YopD_I32K_ and YopD_A270P_ backgrounds from the Δ*yopB*, *yopD* mutant, and YopD_I262P_ mutant backgrounds. Furthermore, IL-1α and IL-10 discriminate the YopD_I262P_ mutant background from the Δ*yopB*, *yopD* mutant background.

A similarly generated volcano plot was established to observe the fold change in cytokines IL-2, IL-6, and GM-CSF levels. In this case, we were not able to identify any particular patterns (Figure 9b). This is a clear indication that the fold change for these three cytokines were indeed subtly different for parent versus parent-MYM, YopD_I32K_ versus YopD_I32K_-MYM, and YopD_A270P_ versus YopD_A270P_-MYM strains. Hence, even though cluster analysis has consistently indicated that the backgrounds of these strains cause a host response that is highly similar, modest differences in IL-2, IL-6, and GM-CSF fold change between them confirm that they are clearly not identical. This corroborates phenotypic differences that were consistently observed in several biological tests (see Figures 1–4, and Supplementary Table S1).

## Discussion

Knowledge is limited concerning the stepwise biogenesis of the T3SS translocon pore and its relationship toward effector protein translocation. Encouragingly, recent mutagenesis studies of the YopD translocator have been able to genetically demarcate discrete functional domains [29,32,33,51,59–61]. This hints at the possibility that YopD mutants could be generated that have targeted deficits in one or a few phenotypes in isolation from all others. This study has extended previous studies of the valuable YopD_I32K_, YopD,_I262P_ or YopD_A270P_ point mutants [30,31] (summarized in Table S1). We characterize relevant mutant types that exhibit well-defined and discrete functional deficits in (1) eukaryotic membrane integration and pore formation, (2) effector translocation and molecular targeting in the eukaryotic cell cytosol, (3) manipulation of host cell responses, and/or (4) survival in murine models of infection. Critically, access to these mutant types creates a set of molecular tools to probe the dynamics of translocon pore biogenesis and function in *Y. pseudotuberculosis.*

It is now possible to *in vitro* purify seemingly intact translocon assemblies that can integrate into artificial and biological membranes [17,18,62–65]. Exogenous addition of *in vitro* purified translocon complexes can reconstitute *in vivo* translocon function by bacteria in contact with eukarotic cells [62]. Advances in super-resolution fluorescence microscopy techniques, and cryo-electron tomography now also permits visualization of translocon structures at the bacteria-host cell contact zone [66,67]. We are currently working toward applying these approaches with the intention to purify the translocon complexes associated with the three YopD_I32K_, YopD_I262P_ or YopD_A270P_ mutants to visualize their conformation, and to reveal translocon protein complexes trapped in a compromised state of assembly. It is envisaged that these studies might help to reveal the molecular arrangement and dynamics of translocon pore assembly in host cell membranes.

A key component of this study was the observation that RAW 264.7 macrophages produced different cytokine profiles in response to various *yopD* mutants with different levels of attenuation. The study was constrained to one time point (4 h post infection), which might mean that additional information outside of this sampling window is missed. This could be relevant considering that we observed herein that the YopD mutants differed in their YopH (early) and YopJ (late) translocation efficiencies, which means that effects ascribed to Yop translocation could be delayed in time for a particular strain. Nevertheless, the utility of the model is not so much in the demonstration of different cytokine profiles but in the fact that these cytokine profiles can provide insight into why the different *yopD* mutants are attenuated in the way that they are. To categorize what the cytokine results could imply, we have listed in Supplementary Table S2 those cytokines that are proinflammatory and those that are anti-inflammatory, and related this to the context of a *Yersinia* infection.

Measuring infected RAW macrophage cytokine profiles, combined with unbiased PCA modeling, could segregate the various *Y. pseudotuberculosis* strains. We observed that IL-1α, IL-1β, and IL-10 play a major part in discriminating the parent, YopD_I32K_ and YopD_A270P_ backgrounds from the Δ*yopB*, *yopD* mutant and YopD_I262P_ mutant backgrounds with respect to the presence and absence of effectors. We also observed that IL-1α and IL-10 discriminate the YopD_I262P_ mutant background from the Δ*yopB*, *yopD* mutant background. Furthermore, despite the parent, YopD_I32K_ and YopD_A270P_ backgrounds sharing multiple similarities that drove a single cluster, the different response of IL-2, IL-6, and GM-CSF very likely reflects the subtle phenotype differences that exist between them, i.e. they are highly similar, but they are not identical. This signature innate immune signaling response represents a “barcode” by which the various mutants could be differentiated. Follow-up experiments need to address what aspect of YopD_I32K_-, YopD_I262P_-, and YopD_A270P_ function constitutes the primary signal governing these specific cytokine responses. We suspect that this signal may be a culmination of subtle differences in the way the variants complex with YopB and engage with the host cell surface, generate local perturbations in the host cell plasma membrane, and pilot internalized Yop effectors to their bona fide molecular targets within the host cell. To identify these processes at the structural level would give greater understanding to biogenesis, function and regulation of the translocon by pathogenic *Yersinia* spp.

We were surprised initially by the increased IL-1β response to infection by parental *Y. pseudotuberculosis* in comparison to infection by the MYM effectorless strain. These data contrast with the finding that bone marrow-derived macrophages pretreated with LPS secreted no IL-1β in response to *Y. pseudotuberculosis* wild type, yet secreted IL-1β in response to an isogenic strain lacking just *yopK* and *yopJ* [58], lacking *yopJ* and *yopM* [68], or the MYM strain lacking all effectors [51]. Hence, a prevailing model has pathogenic *Yersinia* spp. able to induce inflammasome activation through numerous pathways, yet use YopK, YopJ, and YopM to inhibit inflammasome activation and therefore IL-1β secretion [6,7]. It is unclear why our MYM strain does not trigger IL-1β production, although it should mediate inflammasome activation. A reason for this could be that the investigated time point was too early to detect inflammasome-dependent IL-1β production. On the other hand, it should be kept in mind that RAW macrophages are considered deficient for the inflammasome component ASC [69], which would impair the IL-1β response. Hence, this limits the ability to compare and contrast the IL-1β result from RAW 264.7 macrophages to other cells.

Pathogenic *Yersinia* spp. have evolved a variety of strategies to modulate host cell death. In our studies, we used a pan-caspase inhibitor, z-VAD to conclude that *Y. pseudotuberculosis*-induced death of RAW264.7 macrophages is caspase mediated. We anticipate that this is an apoptotic cell death, although we do not distinguish between the Caspase 8- and 10-dependent extrinsic pathway and the caspase 9-dependent intrinsic pathway. Caspase 8-mediated cell death induced by infection with pathogenic *Yersinia* spp. has been reported [54–56]. Yet based on previously published work, treatment of primary BMDMs with z-VAD during infection with pathogenic *Yersinia* spp. leads to caspase-independent cell death via programmed necroptosis [54,55,70,71]. The only time that z-VAD treatment alone protects cells from death is when the programmed necroptosis pathway involving RIPK3/MLKL is ablated genetically or disrupted pharmacologically. We are not aware that the RIPK3 necrosis pathway is attenuated in RAW264.7 macrophages. However, RAW264.7 macrophages are known to have disruption in components of their death machinery, including ASC, and it is not implausible for them to have defects in other components as well. Hence, at this stage, we cannot rule out cell death by necrosis. However, cell death via the caspase 1-dependent pyroptosis pathway is unlikely given the absence of ASC in RAW macrophages [69]. Importantly, our data is in agreement with a previous study showing that pan-caspase inhibitor, z-VAD partially partially blocks *Y. pseudotuberculosis*-induced cell death in BMDMs isolated from C57BL/6 mice [72].

YopB-YopD pore assembly in the immune cell membrane causes an inflammatory response independent of the translocated Yop effectors [33,49–52]. Perhaps this is why a functional pore that is not engaged in the process of Yop effector translocation has evolved the ability to dampen IL-6, IL-10 and RANTES production. The dampening of RANTES production adds meaning to the putative involvement of CCR5 – the RANTES cell-surface receptor – in the biogenesis and function of the YopB-YopD translocon pore [73]. Hence, our YopD point mutants provide meaningful tools to explore the coupling between RANTES production, the cell-surface located CCR5, and YopB-YopD pore assembly.

Finally, the mutant producing YopD_A270P_ was impaired in the ability to resist phagocytosis by macrophages, despite efficiently targeting YopH to focal complexes to dephosphorylate FAK. This suggests that FAK dephosphorylation only is insufficient for *Yersinia* to resist phagocytosis. Since YopH has several additional interaction targets inside the host cell that are mostly associated with focal complex function (*e.g*.: p130cas, Fyb, Paxillin, SKAP-HOM, SLP-76, and PRAM-1) [2–4], it is possible that YopH targeting to one or more of these additional targets is compromised in the YopD_A270P_ mutant. This observation provides another illustration that these YopD point mutants have the possibility to pry apart subtle functions of the translocon pore.

## Materials and Methods

### Bacterial strains and culture conditions

All bacterial strains that were used in this study are listed in Supplementary Table S3. *Y. pseudotuberculosis* YPIII/pIB102 (serotype III) is designated as the parental strain, where the plasmid pIB102 encodes for the Ysc-Yop T3SS. Unless otherwise mentioned, bacteria were normally cultivated in lysogeny broth (LB) in liquid or agar form at either 26°C (*Y. pseudotuberculosis*) or 37°C (*E. coli*) with aeration. *Y. pseudotuberculosis* strains were grown overnight in LB liquid medium supplemented with 2.5 mM CaCl_2_ at 26°C with aeration in preparation for tissue culture monolayer infections. The following day, we diluted the overnight cultures to an optical density of 0.1 at wavelength 600 nm in fresh calcium deficient LB (i.e. supplemented with 5 mM EGTA and 20 mM MgCl_2_). The cultures were first grown with aeration at 26°C for 1 h followed by 37°C for 1 h (or until an OD of ~0.4 at a wavelength of 600 nm was reached). Antibiotics were added if it was required at the final concentrations of carbenicillin (Cb; 100 μg/ml), kanamycin (Km; 50 μg/ml), trimethoprim (Tp; 10 μg/ml) and chloramphenicol (Cm; 25 μg/ml).

## Mutant construction

We utilized several mutagenesis vectors that were all derivatives of the suicide plasmid, pDM4 [74] (Supplementary Table S3) to construct *in cis* site-directed and deletion mutants that were used in this study. For the construction of pMKF004, a DNA fragment was amplified from YPIII/pIB44 [75] with the use of the primer combination of xhoI-ypkA311for (5’-GCGCTCGAGTCAGTAATCTCTTTGGAGCC-3’) xbaI-ypkA1480rev (5’-TGCTCTAGAACTGATCCTTGTCTACTCCC-3’), and following *Xho*I/*Xba*I restriction that was cloned into the linearized pDM4. Plasmid DNA was maintained in *E. coli* DH5αλ*pir*, and then transformed into S17–1λ*pir* for use as the donor strain for conjugal mating with *Y. pseudotuberculosis* recipients. Mutated alleles were introduced into the *Y. pseudotuberculosis* genome by a double cross-over homologous recombination event and the desired genotype was recovered by *sacB*-dependent sucrose sensitivity according to an established protocol [74]. The presence of the desired mutations in the genome of *Y. pseudotuberculosis* was verified by PCR and a sequence analysis of the amplified regions that flanked the mutation.

The effector minus multiple Yop mutant (MYM) background was established from the YPIII/pIB29MEK strain that lacked *yopH, YopM, yopE,* and *yopK* [76]. The pDELJ mutagenesis plasmid was conjugated into this strain to introduce a full-length *yopJ* deletion, which gave rise to YPIII/pIB29MEKJ. The pMKF004 mutagenesis plasmid was then conjugated into this strain to introduce a *ypkA* deletion which gave rise to YPIII/pIB29MEKJA – the MYM mutant. The generation of the various YopD point mutants in the effector minus MYM background was achieved sequentially. The first step involved the establishment of the point mutations in the recipient YPIII/pIB29MEK background that was initiated through conjugation of pSF013 (for YopD_I32K_), pSF003 (YopD_I262P_) and pSF006 (YopD_A270P_), respectively. The following steps involved the introduction of a Δ*yopJ* deletion (via conjugation of pDELJ) into these newly generated YopD point mutant backgrounds, and was finally followed by the introduction of a Δ*ypkA* deletion mutant (via conjugation of pMKF004). The mutagenesis vector pMF498 was conjugated into strain YPIII/pIB29MEKJA to generate the effector minus MYM mutant that lacked both the *yopB* and *yopD* alleles.

### Tissue culture cell lines and preparations for bacterial infections

We used the RAW 264.7 cell line (ATCC® TIB-71™) for the majority of infection experiments. The RAW 264.7 cells were maintained and prepared for *Y. pseudotuberculosis* infection studies in DMEM/high glucose, 4500 mg/L, and these were supplemented by 4.00 mM l-glutamine from HyClone™. The medium was supplemented with 10% (v/v) heat inactivated fetal bovine serum (FBS), from Sigma, at 37°C in 5% CO_2_ and a humidified atmosphere. Bacterial infections were made with the use of growing monolayers that had been passaged 10 times or fewer. Where stated, the HeLa cell line (CRM-CCL-2, ATCC) was used in this study. HeLa cells were maintained and prepared for *Y. pseudotuberculosis* infection studies in DMEM medium supplemented with 10% heat inactivated FBS in 5% CO_2_ and a humidified atmosphere.

Bacteria were grown to an OD_600_ of ~0.4. One milliliter of bacterial culture was transferred to an Eppendorf tube and was centrifuged at maximum speed for 2 min. Supernatant was discarded, the bacterial pellet was washed once with PBS to remove selection antibiotic residue. The pellet was suspended in DMEM medium and used for infection. A multiplicity of infection (MOI) of 10 was used for β-lactamase assay, whereas a MOI of 20 was for the phospho-p38 and NF-κB staining using confocal micorsopy, uptake, LDH cytotoxicity, rypan blue assays, and a MOI of 100 for infections of HeLa cells. Infected cell lines were incubated for designated time points as described for each assay.

### FAK dephosphorylation assay

HeLa cells were grown overnight to semi-confluence in 10 cm diameter tissue culture plates (~4 × 10^6^ cells/plate). Overnight growth medium was removed, and the prepared bacteria suspensions were added to the monolayers to a MOI of 100. Infections were performed at 37^°^C in 5% CO_2_ and a humidified atmosphere for the indicated time periods. The tissue culture plates were placed on ice, the bacteria suspensions were discarded, and the remaining HeLa monolayers were washed twice in ice-cold PBS to terminate the infections. Cells were lysed in ice-cold precipitation buffer (50 mM Tris-HCl pH 7.5, 150 mM NaCl, 1 mM EGTA, 1% NP-40, 0.25% sodium deoxycholate, 1 mM Na_3_VO_4_, 1 mM NaF, protease inhibitor cocktail) [38]. Lysates were cleared by centrifugation at 16,000*g* for 10 min at 4°C. FAK was then immunoprecipitated from the prepared lysates according to protocol [39]. In short, cleared lysates were pre-incubated with Protein G-Sepharose beads (4 Fast Flow, GE Healthcare) coupled to purified mouse IgG (Agrisera AB, Vännäs, Sweden) for 1 h at 4°C. Unbound lysate contents were thereafter incubated with anti-FAK (clone 2A7, EMD Millipore) coupled protein G-Sepharose beads for 3 h at 4°C. Beads were washed twice in a precipitation buffer, and the bound protein was eluted by boiling in a 1× SDS-PAGE loading buffer at 95°C for 5 min. Both cleared lysates and protein fractions immunoprecipitated with anti-FAK were analyzed by SDS-PAGE and Western blotting, probed for the presence of phosphorylated FAK (pFAK) using anti-Phosphotyrosine (clone 4G10, EMD Millipore) and anti-mouse-HRP (GE Healthcare). The same membranes were also probed with anti-GAPDH (MAB374, EMD Millipore). Protein band density was determined from scanned X-ray films with the use of ImageJ [77]. Relative sample pFAK content was derived from the detected GAPDH content as a loading control.

### β-Lactamase assay and live cell fluorescence microscopy

YopE_86_-Bla and YopH_99_-Bla translocation by different strains of *Y. pseudotuberculosis* into RAW 264.7 cells was performed as previously described [31]. Briefly, RAW 264.7 cells were seeded (20,000 cells/well) in 18-well ibidi chamber slide. In parallel overnight cultures of *Yersinia* strains were diluted to an optical density of 0.1 at 600 nm in calcium devoid LB (supplemented with 20 mM MgCl_2_ and 5 mM EGTA) and grown with aeration at 26°C for 1 h, followed by incubation at 37°C for 2 h to induce T3SS. One ml of bacterial culture was normalized to the amount of bacterial cells at an optical density of 600 nm and centrifuged at maximum speed for 2 min. Bacterial pellet was washed once with PBSe and resuspended in DMEM media supplemented with 10% (v/v) heat inactivated foetal bovine serum (FBS). RAW 264.7 cells were infected with the T3SS-induced *Yersinia* strains at a MOI of 10 and centrifuged for 5 min at 200 *g*, to synchronize the infection. Infected RAW 264.7 macrophages were incubated at 37°C in 5% CO_2_ for 1 h. After infection, the cells were labeled with CCF2 AM (Invitrogen) in phosphate buffer saline (PBS) for 1 h at room temperature according to the manufacturer’s recommendations. The translocation efficiency of YopE_86_-Bla or YopH_99_-Bla was determined by live-cell fluorescence microscopy (Nikon Eclipse Ti-E) using a true color camera (Nikon DS-2Fi) fitted with a filter designed specifically for visualization of the β-lactamase reaction (Chroma). Images were captured, and the number of blue and green cells were counted using cell segmentation algorithm, Cellpose [78]. Cellpose diameter function with a value of 17 or 22, and a cyto segmentation model was used for cell segmentation of the acquired images.

### Bacterial uptake assay

Cells were grown on 12 mm diameter glass coverslips in a 24-well plate format at a density of ~2.5 × 10^5^ cells/well. We used various bacterial suspensions to infect cell monolayers to a MOI of 20. Infections were performed at 37°C in 5% CO_2_ and a humidified atmosphere for 30 min. The infected media was then carefully removed and the cell monolayers were gently washed twice with PBS, and then fixed with 4% PFA for 10 min at room temperature. A suspension of rabbit-anti-*Yersinia* prepared in 2.5% (w/v) BSA was applied to the surface of the coverslips and incubated for 30 min at room temperature. After this, the surface-fixed cells were washed twice with PBS, prior to permeabilization with 0.5% Triton X-100 for 5 min at room temperature. Following another round of PBS washes, extracellular bacteria were stained for 1 h in the dark with donkey-anti-rabbit Alexa Flour 568 (Life Technologies) prepared in a solution of 2.5% (w/v) BSA. After incubation, the cells were washed twice with PBS and were then overlaid with the same preparation of rabbit-anti-*Yersinia* in 2.5% (w/v) BSA and incubated for 30 min at room temperature. Following an additional round of PBS washes, surface-fixed cells were incubated for 1 h in the dark with donkey-anti-rabbit Alexa Fluor 488 (Life Technologies). After incubation, the cells were washed twice with PBS and then counterstained for 10 min in the dark with Hoechst (diluted 1:10,000 in PBS). After a series of washes in PBS and a final wash in MilliQ water, the coverslips were mounted on glass slides with anti-fade fluorescence mounting medium (Dako, Denmark). The mounted coverslips were allowed to cure overnight under a fluorescent microscope prior to evaluation, with the use of relevant filters (Alexa Flour 568 λ_Ex_ = 578, Alexa Flour 488 λ_ex_ = 495 nm, Hoechst λ_ex_ = 405).

### Detection of Phospho p38 by western blotting

The infected RAW 264.7 cells cultured in a 6-well plate format were lysed in 200 μl of lysis buffer (50 mM Tris-HCl pH7.4, 150 mM NaCl, 1% Triton X-100, 0.5% sodium deoxycholate and 0.1% SDS). The cell lysates were collected into 50 μl of 4× SDS-PAGE loading buffer [200 mM Tris-HCl, pH 6.8, 8% (w/v) SDS, 0.4% (w/v) bromophenol blue, 40% (v/v) Glycerol, 20% (v/v) β-mercaptoethanol] and were then boiled for 10 min. Proteins were resolved using 12% SDS PAGE, prior to a wet transfer onto PVDF transfer membrane (ATCC®-P). Specific membrane-bound proteins were detected with anti-phospho-p38 MAPK (Thr180/Tyr182, #4511) (D3F9) HyClone™, anti-total p38 MAPK (#9212) Rabbit mAb (Cell Signaling Technology Europe, B.V., Leiden, The Netherlands) or mouse derived anti-glyceraldehyde-3-phosphate dehydrogenase (GAPDH) antibody, clone 6C5 (#MAB374, Merck KGaA, Darmstadt, Germany). Appropriate HRP-conjugated secondary antibodies were purchased from GE Healthcare, and used in combination with Pierce ECL Plus Western Blotting Substrate (ThermoFisher Scientific). Images were resolved on X-ray film using a Immobilon® INDEX 900E NDT-Film processor. ImageJ was used to quantify Phospho-p38 MAPK intensity relative to GAPDH levels.

### Immunofluorescence microscopy

The HeLa or RAW 264.7 cells were infected with *Y. pseudotuberculosis* wild-type or mutant strains. HeLa cells were infected for 1 h at an MOI of 100, while RAW 264.7 cells were infected for 4 h at an MOI of 20. After infection, cells were fixed with 4% paraformaldehyde (PFA) for 30 min at room temperature (RT) followed by permeabilization in Triton X (0.25%) for 20 min at RT. The cells were incubated with primary antibodies, anti-phsopho (pY397)-FAK (BD Biosciences, #611723, 1:100 dilution), anti-phospho (Thr180/Tyr182)-p38 MAPK (Cell Signaling, #9211, 1:100 dilution) or anti-NF-κB (Santa Cruz, #sc -81,932, 1:100 dilution) in 10% FBS/PBS overnight at 4°C. The unbound antibodies were washed three times with PBS. Following this, the cells were incubated with secondary Alexa-488 or Alexa-568 conjugated antibodies (1:200 in 10% FBS/PBS, Molecular Probes, Eugene, USA) at RT for 1 h. Cell nuclei were counterstained with DAPI (Sigma, 1 µM in 10% FBS/PBS) at RT for 5 min, followed by three washes with PBS.

The samples were examined with the use of i) Nikon EZ-C1 confocal microscope (Nikon, Japan) that was equipped with a 60X/1.4 plan Apo λs lens and ii) Leica SP8 inverted confocal system (Leica Microsystems) that was equipped with an HC PL APO 63×/1.40 oil immersion lens. The λ_ex_ = 405 nm was selected for DAPI and λ_ex_ = 488 or λ_ex_ = 561 nm for NF-κB and phospho-p38. Images were captured with the NIS-Elements software (Nikon) or LasX (Leica Microsystems). Images were processed and quantified using ImageJ – Fiji distribution [79]. The total fluorescence intensity (FI) was determined by calculating integrated density (area × FI), which was then corrected against the background.

### Viability of *Y. pseudotuberculosis*-infected eukaryotic cells

For a cell membrane integrity assay based upon trypan blue staining, RAW 264.7 cell monolayers were grown overnight in 6-well plate format at density of ~2 × 10^6^ cells/well. Various bacterial suspensions were used to infect cell monolayers to a MOI of 20. Infections were carried out at 37^°^C in 5% CO_2_ and in a humidified atmosphere up to 7 h post-infection. At designated time points, the bacteria suspensions were discarded and the remaining RAW 264.7 cell monolayers were washed twice in PBS, and then overlaid with 0.4% Trypan blue solution (Sigma-Aldrich) for 5 min, after which the cells were washed once with PBS. Cells with compromised cell membrane were evaluated with a Nikon Eclipse Ti microscope. Data are a representation of percentage of dead cells of 10 fields derived from three biological replica.

To quantitatively measure lactate dehydrogenase (LDH) that was released into the media from damaged cells, the Pierce LDH Cytotoxicity Assay Kit (ThermoFisher Scientific) was used. Briefly, cell monolayers that were pre-treated for 30 min with the pan-caspase inhibitor, z-VAD-fmk (50 mM, Cat#ALX-260-020-M001, Enzo Life Sciences) or untreated controls, were infected with individual bacterial strain to a MOI of 20. At designated time points, cell-free supernatants were harvested and 50 μl volumes transferred to wells of a 96-well plate. The reactions were initiated by adding equal volumes of substrate, and after 30 min, the extent of released LDH was quantitated from an analysis of absorbance at 490 and 680 nm recorded by a Tecan Infinite M200 plate reader (Tecan Trading AG, Männerdorf, Switzerland).

### Cytokine profiling

We grew the RAW 264.7 cell monolayers overnight in a six-well plate format at the density of ~2 × 10^6^ cells/well. Various bacterial suspensions were used to infect cell monolayers to a MOI of 20 in three biological replicates. All infections were carried out at 37°C in 5% CO_2_ and in a humidified atmosphere for 4 h. Exposure to lipopolysaccharide (LPS) used the convenience of commercially available LPS from *Escherichia coli* serotype O111:B4 (Sigma-Aldrich), despite that *Y. pseudotuberculosis* grown at 37°C would likely produce LPS with a different acylation pattern compared to *E. coli*. Nevertheless, a solution of LPS prepared in Milli-Q water was added to the cell monolayers to achieve a final concentration of 1 μg/ml. LPS exposure was carried out at 37°C in 5% CO_2_ and a humidified atmosphere for 4 h. Supernatants were collected in Eppendorf tubes, and were then cleared of contaminating bacteria by a 2-min centrifugation at 16,100*g*. Bacteria free supernatants were collected in fresh tubes and submitted to tebu-bio (tebu-bio SAS, Le Perray-en-Yvelines, France) on dry ice for cytokine profiling analysis with the use of a Q-Plex Mouse Cytokine-Screen (16-plex). Two technical replicates were carried out for each biological replicate.

### PBMCs preparation and bacterial infection

Human PBMCs were isolated from buffy coats of blood obtained from healthy donors by Lymphoprep according to the manufacturer’s instructions (Stemcell Technologies, Cambridge, UK). Briefly, dthe blood was diluted with an equal volume of phosphate buffer saline (PBS). The diluted blood was then transferred to half-volume of Ficoll. Samples were then centrifuged for 30 min at 1500 rpm to create a gradient. After centrifugation, the cells in the white ring layer (PBMCs) were collected and washed with PBS three times to remove the platelets. The freshly prepared PBMCs were resuspended in the DMEM complete media, followed by infection with various strains of *Y. pseudotuberculosis* (MOI of 20) for 4 h. Supernatants were collected and filtered with 0.2 μm syringe filter. TNFα production from human PBMCs under various conditions was detected from biological replicates using human TNF alpha ELISA Kit (ab181421, Abcam), according to manufacturers’ instruction.

### Multivariate modeling and statistical analysis of cytokine data

The total cytokine data included 156 samples and 16 cytokine descriptors (X-data: *n* = 156, *K* = 16). The analysis was performed on three controls (untreated/uninfected, LPS exposure, and on those infected with plasmid cured *Y. pseudotuberculosis*) and 10 test strains (i.e. a total of 13 entries), each with three biological replicates. Two technical replicates were performed for each biological replicate and the analysis was replicated on two different plates. The full data included 2496 data entries with 211 data entries defined as “Incalculable Low” and 97 data entries defined as “Incalculable High.” In order to prepare the data for multivariate analysis (MVA), missing values were exchanged with numerical values. The “Incalculable Low” was exchanged with 0.95 multiplied by the minimum value and the “Incalculable High” was exchanged with 1.05 multiplied by the maximum value, this was performed for each cytokine and in the aspect of the analytical plate. Hence, the missing values were in fact not true missing values since they were detected below or above the calibration ranges. The exchange of missing values was set to be moderate since MVA modeling and statistics would be strongly biased by the introduction of increased variability in the data.

Multivariate analysis is perfectly applicable for modeling data with co-varying structures. The MVA model is based on a set of partial latent variables, calculated for each model component separately. The scores (t) define the relations between the observations and the corresponding loadings (p) define the relations between the variables. Principal component analysis (PCA) is an unsupervised data compression technique, and it is mainly used for overview purposes [80]. PCA is useful for the detection of outliers and for the observation of systematic trends in the data. Orthogonal projections to latent structures – discriminant analysis (OPLS-DA) is a supervised regression technique that is used to assess the maximum separation between classes [81]. OPLS-DA allows for an interpretation of the variable structures related to class differences. The MVA models included the Hotelling’s *t*-squared statistic (t2), that was used to represent the “normality boundaries,” i.e. model limits, defined for the modeled data. The data were pre-treated by UV-scaling, i.e. mean centered and unit variance scaled, for ordinary MVA modeling and left as untreated for secondary multivariate modeling of OPLS-DA effect profiles. The goodness of fit (R2X) is the explained modeled variation and the goodness of prediction (Q2) is the predictive ability of the model, estimated for all MVA models.

Multivariate analysis modeling, both PCA and OPLS-DA, was carried out in SIMCA-P+ version 14.0.0.1359 (Umetrics AB). The independent *t*-test and fold changes, combined into volcano plots, was performed in MATLAB R2017a (Mathworks, Natick, MA, USA).

## Other statistical analyses

All quantifications are visualized median unless otherwise mentioned. For statistical analysis, we used one-way analysis of variance (ANOVA) with Dunnett’s post-test and tested significance at ****p < 0.0001, ***p < 0.001, **p < 0.01, *p < 0.05 when samples were compared to uninfected or parental strain infected cells. Statistical tests were performed using GraphPad Prism version 5.00 for Windows (GraphPad Software, San Diego, California, USA, www.graphpad.com) and using the indicated number of independent experiments and included technical replicates for each experiment. Gaussian distribution of sample population is assumed.

## Supplementary Material

Supplemental Material

## Data Availability

The authors confirm that the data supporting the findings of this study are available within the article and its supplementary material, and are available from the corresponding author on reasonable request.
