# Supplementary material for: Macrophage innate immune responses delineate between defective translocon assemblies produced by Yersinia pseudotuberculosis YopD mutants
Source: Virulence. 2023 Aug 24;14(1):2249790. doi: 10.1080/21505594.2023.2249790 (PMC10461508; doi:10.1080/21505594.2023.2249790)
Supplement: Supplemental Material [file KVIR_A_2249790_SM5467.docx]

**SUPPLEMENTARY INFORMATION**

**Macrophage innate immune responses delineate between defective translocon assemblies produced by *Yersinia pseudotuberculosis* YopD mutants**

Salah I. Farag^1,2^, Monika K. Francis^1,2,§^, Jyoti M. Gurung^1,2^, Sun Nyunt Wai^1,2,3^, Hans Stenlund^4,5^, Matthew S. Francis^1,2,*^ and Aftab Nadeem^1,2,3*^

**Tables**

**Table S1.** Summary of the three phenotypically distinctive YopD mutant classes

**Table S2.** Functional categorization of the cytokine response profile

**Table S3.** Bacterial strains and plasmids used in this study

**Figures**

**Figure S1.** Translocation of YopE_86_-Bla and YopH_99_-Bla into RAW 264.7 macrophages by *Y. pseudotuberculosis* strains expressing different YopD variants.

**Figure S2.** Macrophage cytokine production profiles with no power to discriminate between mutant strains of *Y. pseudotuberculosis*

**Figure S3.** Multivariate analysis of cytokine profiles from control RAW 264.7 cells establish the normality boundary

**Figure S4.** TNFα production from human PBMCs discriminate between mutant strains of *Y. pseudotuberculosis*

**Table S1.** Summary of the three phenotypically distinctive YopD mutant classes

|  | **Parent** | **Δ*yopD* deletion** | **Mutant class 1 (eg.: YopD_I32P_)** | **Mutant class 2 (eg.: YopD_A270P_)** | **Mutant class 3 (eg.: YopD_I262P_)** |
| --- | --- | --- | --- | --- | --- |
| Ca^2+^-dependent regulation of Yops synthesis and secretion^1,2^ | Yes | Impaired | Yes | Yes | Yes |
| RBC haemolysis^1,2^ | Yes | Impaired | Yes | Yes, but smaller pores | Impaired |
| YopD/YopB integration into RBC membranes^1^ | Yes | Impaired (for YopB) | ND | Yes | Impaired |
| Effector translocation | | | | | |
| 1. Cytotoxicity^1,2^ | Yes (˂ 30 mins) | No | Yes (˂ 30 mins) | Yes (˂ 30 mins) | Very delayed (˃ 120 mins) |
| 1. Viability of cell-associated bacteria^1,2^ | Yes | No | Yes | Yes | Impaired |
| 1. Protease protection^1,2^ | Yes | No | Yes | Yes | Impaired |
| 1. Yop::Bla translocation^1^ | Yes (60 to 80% of cells) | No (0 cells) | ND | Yes (60 to 80% of cells) | Severely impaired (1% of cells) |
| 1. FAK dephosphorylation^3^ | Yes | No | Yes | Yes | No |
| 1. % internalised bacteria^3^ | Low (~15% of bacteria) | High (~60% of bacteria) | Low (~20% of bacteria) | Intermediate (~40% of bacteria) | Intermediate (~40% of bacteria) |
| 1. p38 dephosphorylation^3^ | Yes | No | Yes | Yes | No |
| 1. Infected cell death^3^ | Yes (~55% of cells) | No (<5% of cells) | Yes (~50% of cells) | Yes (~55% of cells) | Severely impaired (~15% of cells) |
| Cytokine response profiles^3^ | | | | | |
| 1. IL-1α, IL-1β, IL-10, RANTES and TNF-α | Similar | Different | Similar | Similar | Different |
| 1. IL-2, IL-6 and GM-CSF | Different | Similar | Different | Different | Similar |
| *In vivo* oral infections (mouse weight loss)^1,2^ | | | | | |
| 1. High bacterial inoculum (10^9^ cfu/ml) | 25% loss after 4 days | No weight loss | 25% loss after 6 days | 25% loss after 7 days | No weight loss |
| 1. Medium bacterial inoculum (10^8^ cfu/ml) | 25% loss after 5 days | No weight loss | 25% loss after 8 days | 12.5% loss after 7 days | No weight loss |
| 1. Low inoculum (10^7^ cfu/ml) | 25% loss after 6 days | No weight loss | 12.5% loss after 9 days | No weight loss | No weight loss |

1. Data generated from the study of Costa *et al.,* 2013 [1].
2. Data generated from the study of Costa *et al.,* 2012 [2].
3. Data generated in this study

ND Not determined

Colour coding reflects phenotypic characteristics that are highly (dark green) or partially (light green) like parental (‘wild type’) bacteria or highly (dark red) or partially (light red) like the Δ*yopD* null mutant bacteria.

**Table S2.** Functional categorization of the cytokine response profile

| **Responsive cytokines** | **Functionality** | **Implications during a *Yersinia* infection** |
| --- | --- | --- |
| IL-1α | Pro-inflammatory  Released upon tissue damage and necrotic cell death | Yop-effector dependent inhibition of IL-1α production is expected to reduce pathological inflammation due to infection [3, 4]. The T3S translocon pore can cause tissue damage and necrotic cell death, and this is mitigated by the activity of translocated Yop effectors |
| IL-1β | Pro-inflammatory  Mediated by a two-step process, the first at the level of gene expression that involves NF-κB-mediated pro-cytokine production and the second at the level of maturation involving the caspase-1-dependent inflammasome pathway | Since IL-1β promotes a robust early immune response that enhances host resistance against *Yersinia* infection, the bacteria use several Yop effectors to suppress IL-1β secretion that promotes virulence [5-11].  Note that our IL-1β secretion data with RAW macrophages contradicts several published studies and is not considered to be representative of a true *Yersinia* effect. This has been discussed herein. |
| IL-10 | Immunosuppressive / anti-inflammatory  Involved in expansion of Treg cells and establishment of adaptive immunity | Translocated Yop effectors correlate with IL-10 production, and this enhances *Yersinia* virulence [12-14]. |
| RANTES (CCL5) | A chemokine potently induced by the bacterial infections. During the process of infection RANTES acts as a chemoattractant to monocytes and T lymphocytes. | YopJ has been previously shown to block the activity of RANTES promotor [15]. |
| TNFα | Pro-inflammatory  Mediates pleiotropic effects such as apoptosis, cell proliferation, cytokine production as well as stimulates the microbicidal activity of macrophages and polymorphonuclear neutrophils | Translocated Yop effectors inhibit TNFα production that is caused by exposure of cells to *Yersinia* PAMPs [16-20]. Allows *Yersinia* to establish a systemic infection. |
| IL-2 | Pro-inflammatory and anti-inflammatory  Maintains lymphocyte homeostasis. Is involved in activated T-lymphocyte expansion and differentiation. Limits T-cell hyperactivation via activation-induced cell death and promotes immune tolerance through the development of regulatory T cells | A previous study reported the secretion of IL-2 along with several other cytokines including IL-1β (interleukin-1β), IL-4, IL-5, IL-6, IL-10, IL-12, TNFα, GM-CSF and IFN-γ (interferon-γ) as a cytokine storm in response to Yersinia enterocolitica infections. The levels of these cytokines in Y. enterocolitica infected mice reached peak values in a time dependent manner, followed by a sudden decrease in their secretion later during the infection process [4]. |
| IL-6 | Proinflammatory  Pleiotropic, but contributes to acquired immunity by stimulating antibody production and effector T-cell development and attracts neutrophils to infection sites. Can also promote differentiation or proliferation of several nonimmune cells. | *Yersinia* with a functional translocon pore can suppress IL-6 production, independent of the presence of translocated Yop effectors. Since IL-6 is necessary to clear *Yersinia* infections, suppression of IL-6 levels allows *Yersinia* to establish a systemic infection [4, 21, 22]. |
| GM-CSF | Proinflammatory and hemopoietic growth factor  Pleiotropic, but major role in proliferation and differentiation of bone marrow progenitors into granulocytes and macrophages. | There is difficulty in substantiating the impact of GM-CSF on survivability to *Yersinia* infection. It appears that one or more translocated Yop effectors inhibit GM-CSF production that is caused by initial adhesion of the bacteria to epithelial cells and or in response to PAMP exposure [4, 23]. As GM-CSF is a strong chemoattractant and hemopoietic growth factor, suppression of this cytokine is anticipated to reduce immune cell differentiation, cellular migration, and ultimately cellular mediated immunity, although this is not well-established experimentally. |

**Table S3.** Bacterial strains and plasmids used in this study

| **Strains** | **Relevant genotype or phenotype** | **Source/Reference** |
| --- | --- | --- |
| *E. coli* |  |  |
| DH5 | F^−^, *recA*1, *endA*1, *hsdR*17, *supE*44, *thi*-1, *gyrA*96, *relA*1 | [24] |
| S17-1λ*pir* | *recA*, *thi*, *pro*, *hsdR^-^M^+^,* Sm^R^, <RP4:2-Tc:Mu:Ku:Tn7>Tp^R^ | [25] |
| *Y. pseudotuberculosis* |  |  |
| YPIII/pIB102 | pIB102, *yadA*::Tn5, Km^R^ (parent) | Hans Wolf-Watz |
| YPIII | Plasmid-cured | [26] |
| YPIII/pIB44 | pIB102, ypkA in-frame deletion, Km^R^ | [27] |
| YPIII/pIB232 | pIB102, yopJ in-frame deletion, Km^R^ | [28] |
| YPIII/pIB621 | pIB102, yopD in-frame deletion of codons 4 to 303, Km^R^ | [29] |
| YPIII/pIB619 | pIB102, *yopB* and yopD in-frame deletion, Km^R^ | [30] |
| YPIII/pIB29MEK | pIB29, YopH, YopM, YopE, and YopK deletions, Km^R^ | [31] |
| YPIII/pIB29MEKJ | pIB29, YopH, YopM, YopE, YopK and YopJ deletions, Km^R^ | This study |
| YPIII/pIB29MEKJA | pIB29MEKJ, *ypkA* in-frame deletion of codons 207 to 388, Km^R^ | This study |
| YPIII/pIB29MEKJABD | pIB29MEKJA, *yopB* and yopD in-frame deletion, Km^R^ | This study |
| YPIII/pIB60501 | pIB102, *yopD* containing the codon substitution I32K, Km^R^ | [2] |
| YPIII/pIB29MEK60501 | pIB29MEK, *yopD* containing the codon substitution I32K, Km^R^ | This study |
| YPIII/pIB29MEKJ60501 | pIB29MEKJ, *yopD* containing the codon substitution I32K, Km^R^ | This study |
| YPIII/pIB29MEKJA60501 | pIB29MEKJA, *yopD* containing the codon substitution I32K, Km^R^ | This study |
| YPIII/pIB63301 | pIB102, *yopD* containing the codon substitution I262P, Km^R^ | [1] |
| YPIII/pIB29MEK63301 | pIB29MEK, *yopD* containing the codon substitution I262P, Km^R^ | This study |
| YPIII/pIB29MEKJ63301 | pIB29MEKJ, *yopD* containing the codon substitution I262P, Km^R^ | This study |
| YPIII/pIB29MEKJA63301 | pIB29MEKJA, *yopD* containing the codon substitution I262P, Km^R^ | This study |
| YPIII/pIB63304 | pIB102, *yopD* containing the codon substitution A270P, Km^R^ | [1] |
| YPIII/pIB29MEK63304 | pIB29MEK, *yopD* containing the codon substitution A270P, Km^R^ | This study |
| YPIII/pIB29MEKJ63304 | pIB29MEKJ, *yopD* containing the codon substitution A270P, Km^R^ | This study |
| YPIII/pIB29MEKJA63304 | pIB29MEKJA *yopD* containing the codon substitution A270P, Km^R^ | This study |
| YPIII/pIB615 | pIB102, *yopB* in-frame deletion of codons 13–399, Km^R^ | [32] |
| YPIII/pIB102-YopE-Bla | pIB102, expressing YopE_86_-Bla_24–286_, Cml^R^, Km^R^ | This study |
| YPIII/pIB615-YopE-Bla | pIB615, expressing YopE_86_-Bla_24–286_, Cml^R^, Km^R^ | This study |
| YPIII/pIB60501-YopE-Bla | pIB60501, expressing YopE_86_-Bla_24–286_, Cml^R^, Km^R^ | This study |
| YPIII/pIB63301-YopE-Bla | pIB63301, expressing YopE_86_-Bla_24–286_, Cml^R^, Km^R^ | [1] |
| YPIII/pIB63304-YopE-Bla | pIB63304, expressing YopE_86_-Bla_24–286_, Cml^R^, Km^R^ | [1] |
|  |  |  |
| YPIII/pIB102-YopH-Bla | pIB102, expressing YopH_99_-Bla_24–286_, Cml^R^, Km^R^ | This study |
| YPIII/pIB615-YopH-Bla | pIB615, expressing YopH_99_-Bla_24–286_, Cml^R^, Km^R^ | This study |
| YPIII/pIB60501-YopH-Bla | pIB60501, expressing YopH_99_-Bla_24–286_, Cml^R^, Km^R^ | This study |
| YPIII/pIB63301-YopH-Bla | pIB63301, expressing YopH_99_-Bla_24–286_, Cml^R^, Km^R^ | [1] |
| YPIII/pIB63304-YopH-Bla | pIB63304, expressing YopH_99_-Bla_24–286_, Cml^R^, Km^R^ | [1] |
| **Plasmids** |  |  |
| pDM4 | Suicide plasmid carrying *sacBR*, Cm^R^ | Debra Milton |
| pSF013 | pDM4 containing a XhoI/XbaI 470 bp overlap PCR product of the allele for YopD_I32K_, Cm^R^ | [2] |
| pSF003 | pDM4 containing a XhoI/XbaI 558 bp overlap PCR product of the allele for YopD_I262P_, Cm^R^ | [1] |
| pSF006 | pDM4 containing a XhoI/XbaI 558 bp overlap PCR product of the allele for YopD_A270P_, Cm^R^ | [1] |
| pMF498 | pDM4 containing a SpeI/XbaI overlap PCR product of the allele for Δ*yopB, yopD*, Cm^R^ | [30] |
| pMF024 | pDM4 containing a SpeI/XbaI 422 bp overlap PCR product of the allele for YopD_Δ4-303_, Cm^R^ | [29] |
| pDELJ | pDM4 containing a PCR amplified DNA fragment of the allele for YopJ_Δ5-792_, Cm^R^ | Britt-Marie Kihlberg |
| pNQ705-E-Bla | pNQ705 containing a *yopE*_6–86_-β-lactamase fusion, Cm^R^ | [1] |
| pNQ705-H-Bla | pNQ705 containing a *yopH*_6–99_-β-lactamase fusion, Cm^R^ | [1] |
| pMKF004 | pDM4 containing a XhoI/XbaI 558 bp overlap PCR product of the allele for YpkA_Δ207-388_, Cm^R^ | This study |


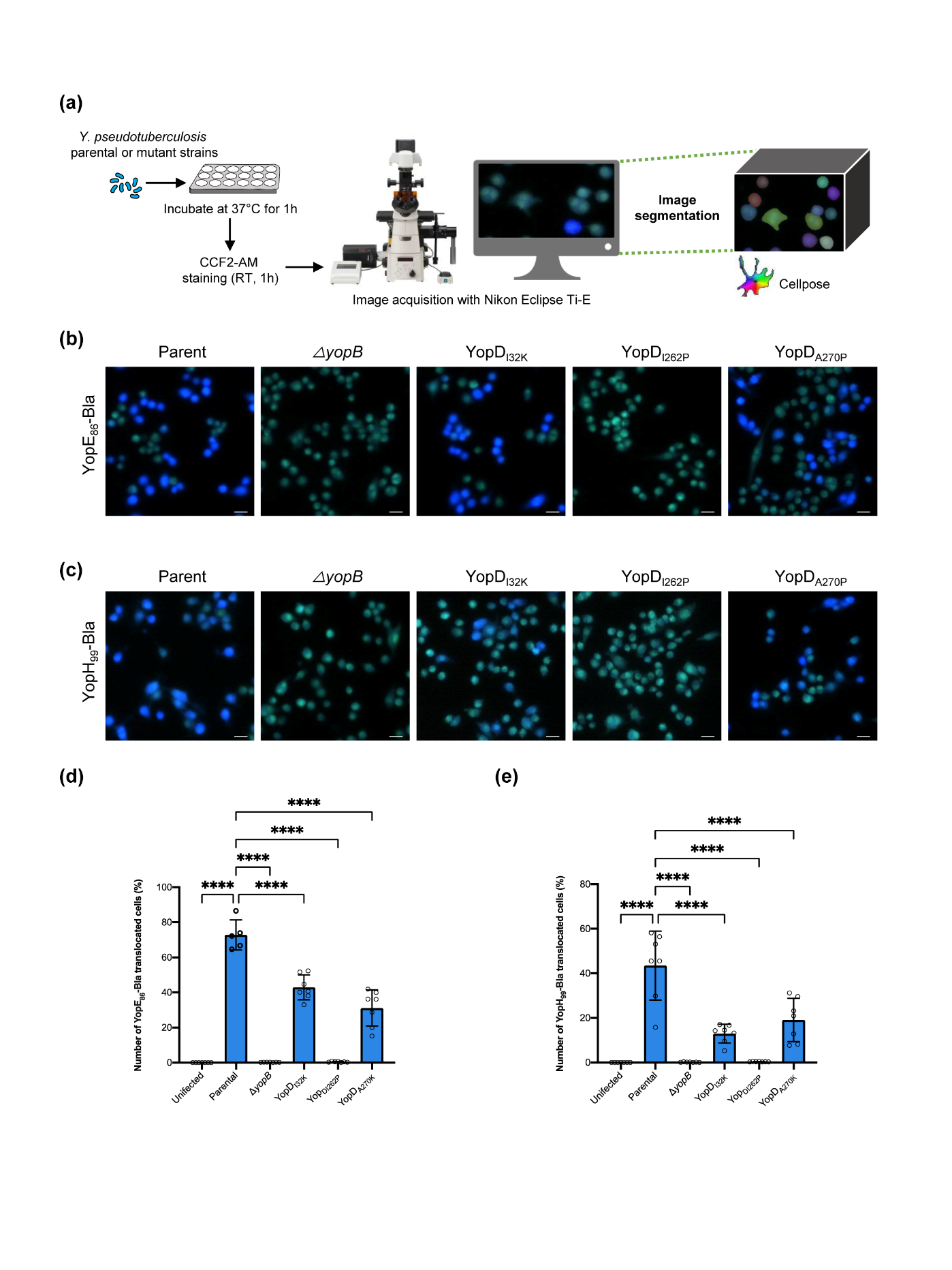


**Figure S1.** Translocation of YopE_86_-Bla and YopH_99_-Bla into RAW 264.7 macrophages by *Y. pseudotuberculosis* strains expressing different YopD variants. The *yopE_6–_*_86_-*bla_24–286_* or *yopH_6–_*_99_-*bla_24–286_* fusions were introduced *in cis* into *Y. pseudotuberculosis* strains expressing the indicated YopD variants, followed by infecting the RAW 264.7 macrophages for 1 h at an MOI of 10. Next, the cells were labelled with CCF2-AM, followed by live cell microscopy. Translocation of the fusion proteins into the infected cells results in an increase in the number of cells with blue fluorescence. (**a**) Schematic workflow used for RAW 264.7 macrophages infection with variants of *Y. pseudotuberculosis*, followed by cell labelling with CCF2-AM dye, image acquisition, and cell segmentation using Cellpose. (**b-c**) Representative images of YopE_86_-Bla or YopH_99_-Bla into RAW 264.7 macrophages infected with fusion-bearing strains of *Y. pseudotuberculosis* encoding the indicated YopD variants. (**d-e**) Histogram indicates percentage of blue cells after infection with the *Y. pseudotuberculosis* strains bearing either YopE_86_-Bla or YopH_99_-Bla. Data in the histograms are representative from 5-7 random microscopy fields of view (382-2555 cells per field of view); bar graphs show mean ± s.d. Significance was determined using a one-way analysis of variance (ANOVA) with Dunnett’s post-test against parent strain of *Y. pseudotuberculosis*. **** *p* < 0.0001, ** *p* < 0.01, * *p* < 0.05. or ns = not significant.


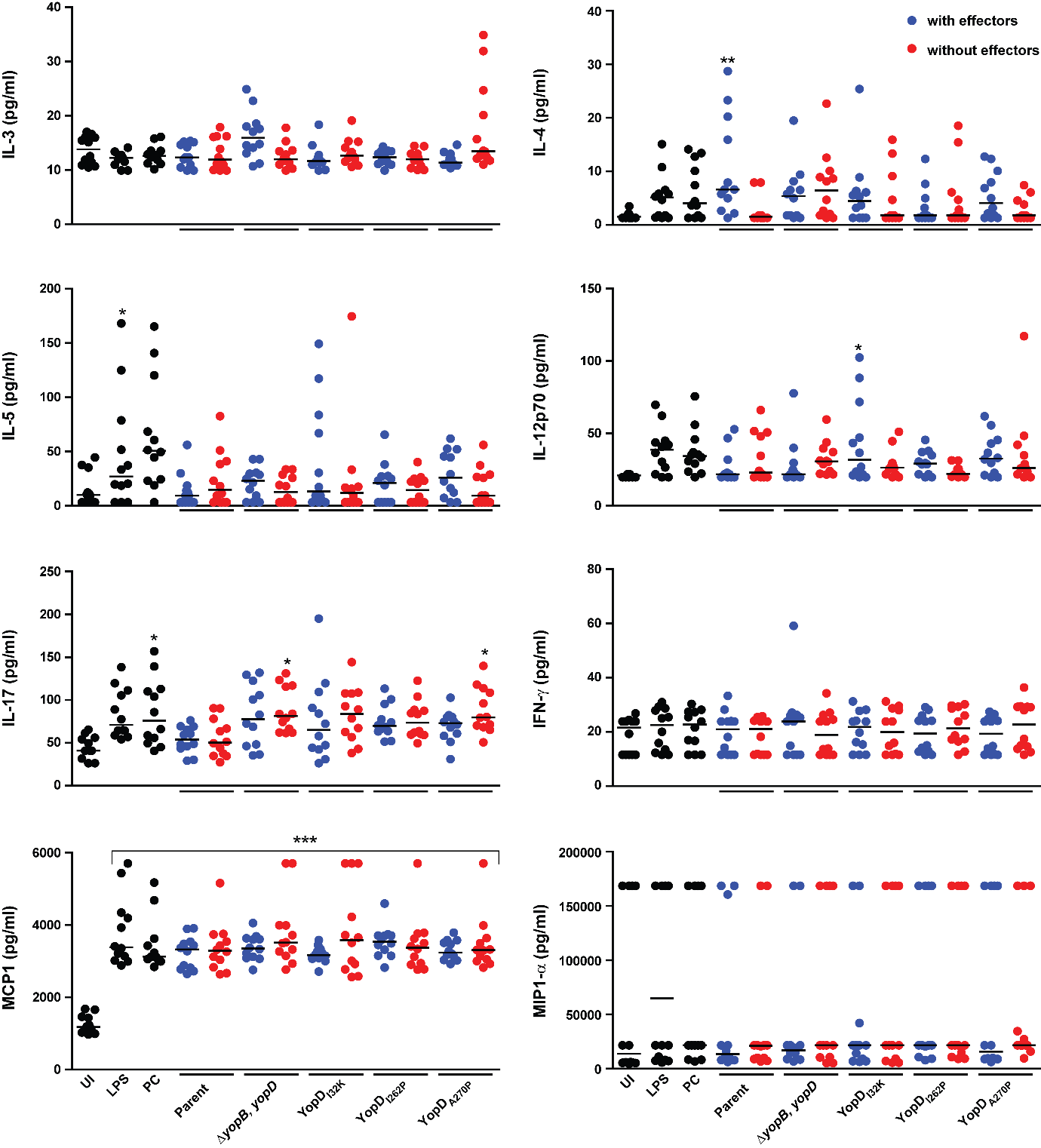


**Figure S2.** Macrophage cytokine production profiles with no power to discriminate between mutant strains of *Y. pseudotuberculosis.* See the legend to Figure 5 in the main text for relevant details. Significance was determined from biological replicates using a two-tailed, unpaired Student's t test. The triple asterisk (***), double asterisk (**) and single asterisk (*) reflects the degree of significant difference with *p* < 0.001, *p* < 0.01 and *p* < 0.05, respectively.


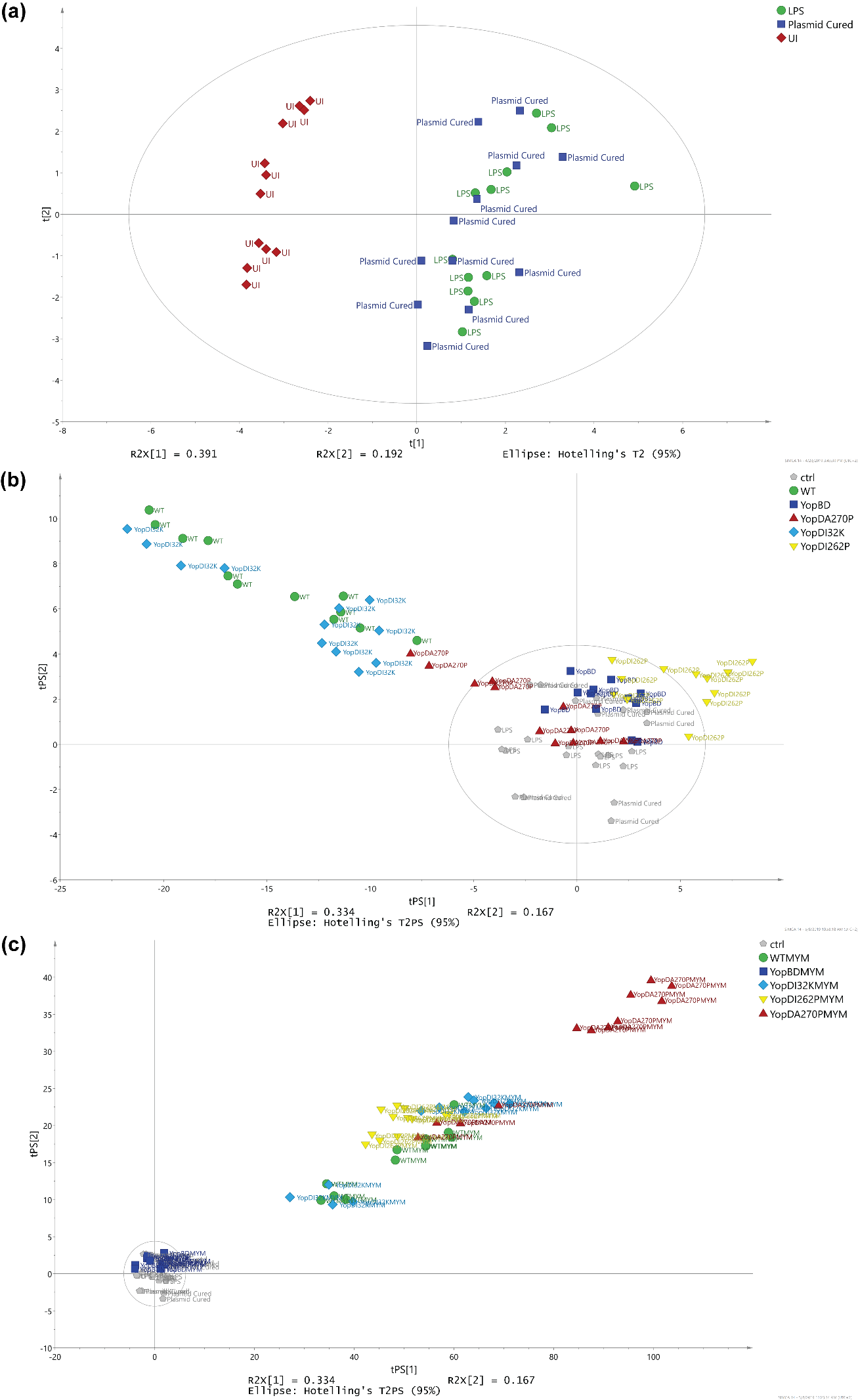


**Figure S3.** Multivariate analysis of cytokine profiles from RAW 264.7 cells establish the normality boundary, with Hotelling's t-squared statistics (t2)(95%) defining the normality limits for the models. (**a**) Displayed are the scores (t1/t2) plot for the PCA model [N=36, K=16, 2 comp., R2X(cum)=0.584, Q2(cum)=0.409] for the control samples. The primary control conditions are represented by cells exposed to either 1 mg/ml purified *E. coli* lipopolysaccharide (LPS) or to *Y. pseudotuberculosis* YPIII that has been cured of the virulence plasmid encoding for the Ysc-Yop T3SS (Plasmid Cured), and by uninfected cells (UI). The LPS (green circles) and Plasmid Cured (blue squares) indicated to be highly similar, compared with the UI (red diamonds). (**b**) Scores (t1/t2) plot for the PCA model (N=24, K=16, 2 comp., R2X(cum)=0.501, Q2(cum)=0.117) fitted for the control (ctrl – grey symbols) samples LPS and Plasmid Cured. The five strains WT (YPIII/pIB102 – parent; green circles), YopBD (YPIII/pIB619 - Δ*yopB*ΔyopD full-length null mutant; dark blue squares), YopD_I32K_ (YPIII/pIB60501 - *yopD* containing the codon substitution I32K; light blue diamonds), YopD_I262P_ (YPIII/pIB63301 - *yopD* containing the codon substitution I262P – yellow triangles) and YopD_A270P_ (YPIII/pIB63304 - *yopD* containing the codon substitution A270P; red triangles) (N=60, K=16) were predicted into the model. All the YopBD samples ended up clearly within the model limits indicating that the YopBD strain was inseparable from the controls. Also, the main part of the YopD_A270P_ samples ended up within model limits. Both the WT samples and the YopD_I32K_ samples obviously deviated from the controls. The main part of the YopD_I262P_ samples indicated to deviate, however not similar compared with the WT and YopD_I32K_ samples. (**c**) Scores (t1/t2) plot for the PCA model (N=24, K=16, 2 comp., UV-scaled, R2X(cum)=0.501, Q2(cum)=0.117) fitted for the LPS and Plasmid Cured control samples. The five multiple *yop* mutant (MYM – lacking *yopH, yopM, yopE, yopK, yopJ* and *ypkA*) strains – WTMYM (YPIII/pIB29MEKJA – parent), YopBDMYM (YPIII/pIB29MEKJABD – MYM also lacking *yopB* and *yopD*), YopD_I32K_MYM (YPIII/pIB29MEKJA60501 – MYM with *yopD* containing the codon substitution I32K), YopD_I262P_MYM (YPIII/pIB29MEKJA63301 – MYM with *yopD* containing the codon substitution I262P) and YopD_A270P_MYM (YPIII/pIB29MEKJA63304 – MYM with *yopD* containing the codon substitution A270P) – (N=60, K=16) were predicted into the model. All the YopBDMYM samples ended up clearly within the model limits indicating that the YopBDMYM strain could not be differentiated from the controls. The other strains – *i.e.*: WTMYM, YopD_I32K_MYM, YopD_I262P_MYM, and YopD_A270P_MYM, were clearly different compared to the controls. The YopD_A270P_MYM appeared as the most deviating strain. Due to the common plot directions, this deviation is mainly caused by total abundance differences. The YopD_I262P_MYM ended up in close relation with the others, however, a small shift in directions was indicated.

**
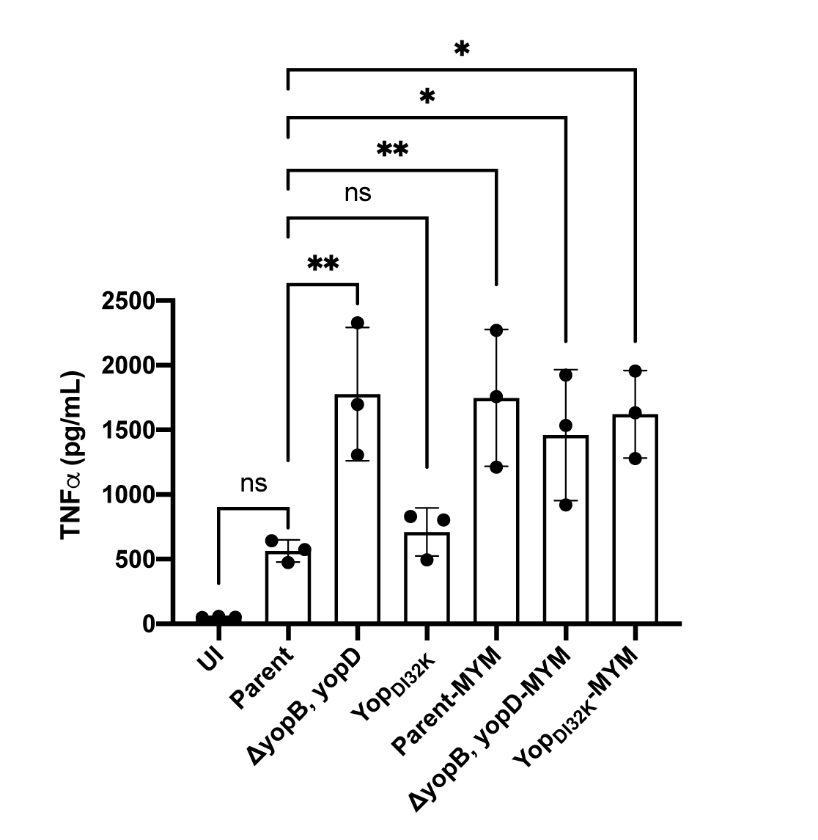
**

**Figure S4.** TNFα production from human PBMCs discriminate between mutant strains of *Y. pseudotuberculosis.* Human PBMCs were infected with variants of *Y. pseudotuberculosis* for 4 hours at an MOI of 20. Cleared culture supernatants were collected and sterile filtered followed by quantification of TNFα production. Data points in histogram indicates data from three independent replicates. Significance was determined from replicates using one-way analysis of variance (ANOVA) with Dunnett’s post-test against cells infected with parent strain of *Y. pseudotuberculosis*. ** *p* < 0.01, * *p* < 0.05. or ns = not significant.

**REFERENCES**

1. Costa TR, Amer AA, Farag SI, Wolf-Watz H, Fallman M, Fahlgren A, et al. Type III secretion translocon assemblies that attenuate *Yersinia* virulence. Cell Microbiol 2013; 15:1088-110.

2. Costa TR, Amer AA, Fallman M, Fahlgren A, Francis MS. Coiled-coils in the YopD translocator family: A predicted structure unique to the YopD N-terminus contributes to full virulence of *Yersinia pseudotuberculosis*. Infect Genet Evol 2012; 12:1729-42.

3. Dube PH, Revell PA, Chaplin DD, Lorenz RG, Miller VL. A role for IL-1 alpha in inducing pathologic inflammation during bacterial infection. Proc Natl Acad Sci U S A 2001; 98:10880-5.

4. Wang X, Gu W, Qiu H, Xia S, Zheng H, Xiao Y, et al. Comparison of the cytokine immune response to pathogenic *Yersinia enterocolitica* bioserotype 1B/O:8 and 2/O:9 in susceptible BALB/C and resistant C57BL/6 mice. Mol Immunol 2013; 55:365-71.

5. Brodsky IE, Palm NW, Sadanand S, Ryndak MB, Sutterwala FS, Flavell RA, et al. A *Yersinia* effector protein promotes virulence by preventing inflammasome recognition of the type III secretion system. Cell Host Microbe 2010; 7:376-87.

6. Casson CN, Copenhaver AM, Zwack EE, Nguyen HT, Strowig T, Javdan B, et al. Caspase-11 activation in response to bacterial secretion systems that access the host cytosol. PLoS Pathog 2013; 9:e1003400.

7. Chung LK, Park YH, Zheng Y, Brodsky IE, Hearing P, Kastner DL, et al. The *Yersinia* virulence factor YopM hijacks host kinases to inhibit type III effector-triggered activation of the pyrin inflammasome. Cell Host Microbe 2016; 20:296-306.

8. LaRock CN, Cookson BT. The *Yersinia* virulence effector YopM binds caspase-1 to arrest inflammasome assembly and processing. Cell Host Microbe 2012; 12:799-805.

9. Ratner D, Orning MP, Proulx MK, Wang D, Gavrilin MA, Wewers MD, et al. The *Yersinia* pestis effector YopM inhibits pyrin inflammasome activation. PLoS Pathog 2016; 12:e1006035.

10. Sivaraman V, Pechous RD, Stasulli NM, Eichelberger KR, Miao EA, Goldman WE. *Yersinia pestis* activates both IL-1beta and IL-1 receptor antagonist to modulate lung inflammation during pneumonic plague. PLoS Pathog 2015; 11:e1004688.

11. Shin H, Cornelis GR. Type III secretion translocation pores of *Yersinia enterocolitica* trigger maturation and release of pro-inflammatory IL-1beta. Cell Microbiol 2007; 9:2893-902.

12. McPhee JB, Mena P, Zhang Y, Bliska JB. Interleukin-10 induction is an important virulence function of the *Yersinia pseudotuberculosis* type III effector YopM. Infect Immun 2012; 80:2519-27.

13. McPhee JB, Mena P, Bliska JB. Delineation of regions of the *Yersinia* YopM protein required for interaction with the RSK1 and PRK2 host kinases and their requirement for interleukin-10 production and virulence. Infect Immun 2010; 78:3529-39.

14. Berneking L, Schnapp M, Rumm A, Trasak C, Ruckdeschel K, Alawi M, et al. Immunosuppressive *Yersinia* effector YopM binds DEAD Box Helicase DDX3 to control ribosomal S6 kinase in the nucleus of host cells. PLoS Pathog 2016; 12:e1005660.

15. Zhou L, Tan A, Hershenson MB. *Yersinia* YopJ inhibits pro-inflammatory molecule expression in human bronchial epithelial cells. Respir Physiol Neurobiol 2004; 140:89-97.

16. Boland A, Cornelis GR. Role of YopP in suppression of tumor necrosis factor alpha release by macrophages during *Yersinia* infection. Infect Immun 1998; 66:1878-84.

17. Lemaitre N, Sebbane F, Long D, Hinnebusch BJ. *Yersinia* pestis YopJ suppresses tumor necrosis factor alpha induction and contributes to apoptosis of immune cells in the lymph node but is not required for virulence in a rat model of bubonic plague. Infect Immun 2006; 74:5126-31.

18. Palmer LE, Hobbie S, Galán JE, Bliska JB. YopJ of *Yersinia pseudotuberculosis* is required for the inhibition of macrophage TNF-alpha production and downregulation of the MAP kinases p38 and JNK. Mol Microbiol 1998; 27:953-65.

19. Ruckdeschel K, Harb S, Roggenkamp A, Hornef M, Zumbihl R, Kohler S, et al. *Yersinia enterocolitica* impairs activation of transcription factor NF-kappaB: involvement in the induction of programmed cell death and in the suppression of the macrophage tumor necrosis factor alpha production. J Exp Med 1998; 187:1069-79.

20. Auerbuch V, Golenbock DT, Isberg RR. Innate immune recognition of *Yersinia pseudotuberculosis* type III secretion. PLoS Pathog 2009; 5:e1000686.

21. Dube PH, Handley SA, Lewis J, Miller VL. Protective role of interleukin-6 during *Yersinia enterocolitica i*nfection is mediated through the modulation of inflammatory cytokines. Infect Immun 2004; 72:3561-70.

22. Matteoli G, Fahl E, Warnke P, Muller S, Bonin M, Autenrieth IB, et al. Role of IFN-gamma and IL-6 in a protective immune response to *Yersinia enterocolitica* in mice. BMC Microbiol 2008; 8:153.

23. Kampik D, Schulte R, Autenrieth IB. *Yersinia enterocolitica* invasin protein triggers differential production of interleukin-1, interleukin-8, monocyte chemoattractant protein 1, granulocyte-macrophage colony-stimulating factor, and tumor necrosis factor alpha in epithelial cells: implications for understanding the early cytokine network in *Yersinia* infections. Infect Immun 2000; 68:2484-92.

24. Hanahan D. Techniques for transformation of *E. coli*. In: Glover DM, ed. DNA Cloning A practical approach. Oxford, United Kingdom: IRL Press Ltd, 1985:109-36.

25. Simon R, Priefer U, Pühler A. A broad host range mobilisation system for in vivo genetic engineering: transposon mutagenesis in Gram negative bacteria. Nature Biotechnology 1983; 1:784-91.

26. Bölin I, Norlander L, Wolf-Watz H. Temperature-inducible outer membrane protein of *Yersinia pseudotuberculosis* and *Yersinia enterocolitica* is associated with the virulence plasmid. Infect Immun 1982; 37:506-12.

27. Galyov EE, Hakansson S, Forsberg A, Wolf-Watz H. A secreted protein kinase of *Yersinia pseudotuberculosis* is an indispensable virulence determinant. Nature 1993; 361:730-2.

28. Galyov EE, Håkansson S, Wolf-Watz H. Characterization of the operon encoding the YpkA Ser/Thr protein kinase and the YopJ protein of *Yersinia pseudotuberculosis*. J Bacteriol 1994; 176:4543-8.

29. Francis MS, Wolf-Watz H. YopD of *Yersinia pseudotuberculosis* is translocated into the cytosol of HeLa epithelial cells: evidence of a structural domain necessary for translocation. Mol Microbiol 1998; 29:799-813.

30. Edqvist PJ, Bröms JE, Betts HJ, Forsberg Å, Pallen MJ, Francis MS. Tetratricopeptide repeats in the type-III-secretion chaperone, LcrH: their role in substrate binding and secretion. Molecular Microbiology 2006; 59:31-44.

31. Håkansson S, Galyov EE, Rosqvist R, Wolf-Watz H. The *Yersinia* YpkA Ser/Thr kinase is translocated and subsequently targeted to the inner surface of the HeLa cell plasma membrane. Mol Microbiol 1996; 20:593-603.

32. Broms JE, Forslund AL, Forsberg A, Francis MS. Dissection of homologous translocon operons reveals a distinct role for YopD in type III secretion by *Yersinia pseudotuberculosis*. Microbiology (Reading) 2003; 149:2615-26.
